# Supplementary material for: A comparison of DNA methylation detection between HiFi sequencing and whole genome bisulfite sequencing in monozygotic twins with Down syndrome
Source: PLoS One. 2025 Aug 5;20(8):e0329593. doi: 10.1371/journal.pone.0329593 (PMC12324119; doi:10.1371/journal.pone.0329593)
Supplement: S1 Table — (PDF) [file pone.0329593.s001.pdf]

S1 Table. Overview of sequencing results from WGBS (wg-blimp) and HiFi WGS data.

	Twin A		Twin B	
	WGBS	HiFi WGS	WGBS	HiFi WGS
Average depth of coverage	24.03X	32.88X	23.34X	34.09X
Number of reads	608691720	6065063	611041232	6580083
Mean read length	151	14878	151	14595
GC content (mean/read)	21.49%	41.67%	21.53%	41.49%
Percent read mapped	99.96%	100%	99.96%	100%
Total CpG sites (depth ≥ 4)	22997803	28656944	22973234	28667271
Methylated CpG sites*	20992773	24134696	20865705	24066630
Overlapping mCs	19985522 (79.5 %)		19842868 (79.1 %)	

* CpG sites with methylation level ≥ 50% and read coverage ≥ 4
