# Supplementary material for: A comparison of DNA methylation detection between HiFi sequencing and whole genome bisulfite sequencing in monozygotic twins with Down syndrome
Source: PLoS One. 2025 Aug 5;20(8):e0329593. doi: 10.1371/journal.pone.0329593 (PMC12324119; doi:10.1371/journal.pone.0329593)
Supplement: S2 Table — (PDF) [file pone.0329593.s002.pdf]

S2 Table. Comparison of WGBS methylation and bisulfite conversion metrics between wg-blimp and Bismark pipelines.

	Twin A		Twin B	
	WG blimp	Bismark	WG blimp	Bismark
Total CpG sites (depth ≥ 4)	22997803	18884441	22973234	18350569
**Percent methylation (across genome)**				
CpG methylation (%)	84.44%	85.4%	83.73%	84.8%
CHG methylation (%)	2.44%	2.4%	2.42%	2.4%
CHH methylation (%)	2.65%	2.8%	2.64%	2.8%
Bisulfite conversion efficiency (%) (100 – % CHH methylation)	97.35	97.2	97.36	97.2
Methylated CpG sites*	20992773	17501359	20865705	16902166
Overlapping mCs	17105751 (80 %)		16495816 (77.5 %)	

* CpG sites with methylation level ≥ 50% and read coverage ≥ 4
