# Supplementary material for: A comparison of DNA methylation detection between HiFi sequencing and whole genome bisulfite sequencing in monozygotic twins with Down syndrome
Source: PLoS One. 2025 Aug 5;20(8):e0329593. doi: 10.1371/journal.pone.0329593 (PMC12324119; doi:10.1371/journal.pone.0329593)
Supplement: S3 Table — (PDF) [file pone.0329593.s003.pdf]

**S3 Table. Comparison of CpG methylation statistics between Bismark (WGBS) and HiFi WGS.**

| | Twin A | | Twin B | |
|---|---|---|---|---|
| | Bismark | HiFi WGS | Bismark | HiFi WGS |
| Total CpG sites (dp ≥ 4) | 18884441 | 28656944 | 18350569 | 28667271 |
| Methylated CpG sites* | 17501359 | 24134696 | 16902166 | 24066630 |
| Overlapping mCs | 16587186 (66.2 %) | | 15998678 (64.1 %) | |

* CpG sites with methylation level ≥ 50% and read coverage ≥ 4
