# Supplementary material for: A comparison of DNA methylation detection between HiFi sequencing and whole genome bisulfite sequencing in monozygotic twins with Down syndrome
Source: PLoS One. 2025 Aug 5;20(8):e0329593. doi: 10.1371/journal.pone.0329593 (PMC12324119; doi:10.1371/journal.pone.0329593)
Supplement: S4 Table — (PDF) [file pone.0329593.s004.pdf]

S4 Table. Comparison of mC detection at 50% and 80% methylation thresholds across platforms.

	Twin A			Twin B		
	HiFi WGS	WGBS (wg-blimp)	WGBS (Bismark)	HiFi WGS	WGBS (wg-blimp)	WGBS (Bismark)
mCs (≥50% methylation)	24134696	20992773	17501359	24066630	20865705	16902166
mCs (≥80% methylation)	20054751	17868738	14711065	19901758	17554322	14048725
Overlap with 50% (%)	(83.10%)	(85.12%)	(84.06%)	(82.69%)	(84.13%)	(83.12%)
