# Supplementary material for: A comparison of DNA methylation detection between HiFi sequencing and whole genome bisulfite sequencing in monozygotic twins with Down syndrome
Source: PLoS One. 2025 Aug 5;20(8):e0329593. doi: 10.1371/journal.pone.0329593 (PMC12324119; doi:10.1371/journal.pone.0329593)
Supplement: S1 Fig — The plots illustrate how the number of detected CpG sites varies with increasing coverage thresholds for (A) HiFi WGS, (B) WGBS (wg-blimp), (C) WGBS (Bismark). (PDF) [file pone.0329593.s005.pdf]

(A)

Twin A

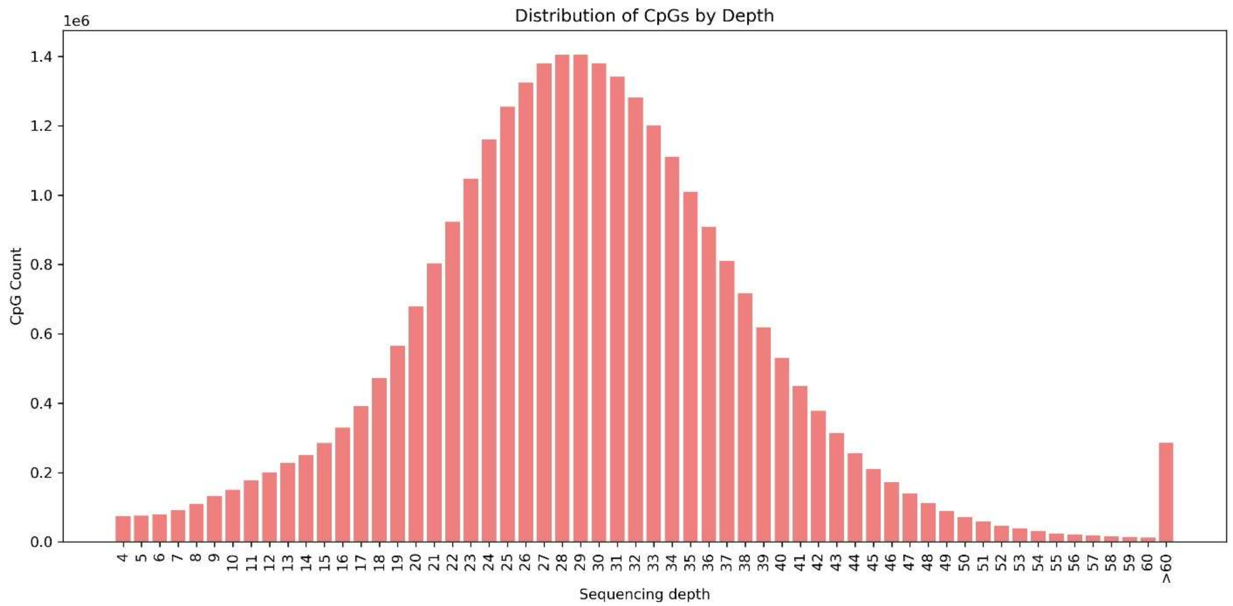

Distribution of CpGs by Depth
1e6
CpG Count
Sequencing depth

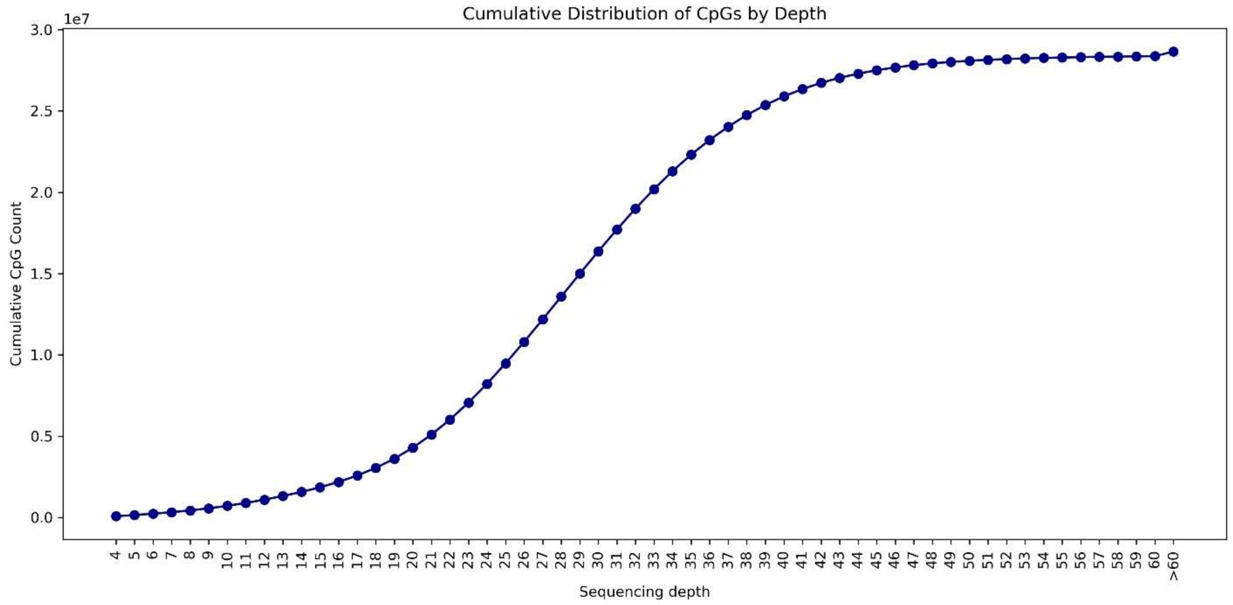

Cumulative Distribution of CpGs by Depth
1e7
Cumulative CpG Count
Sequencing depth

Twin B

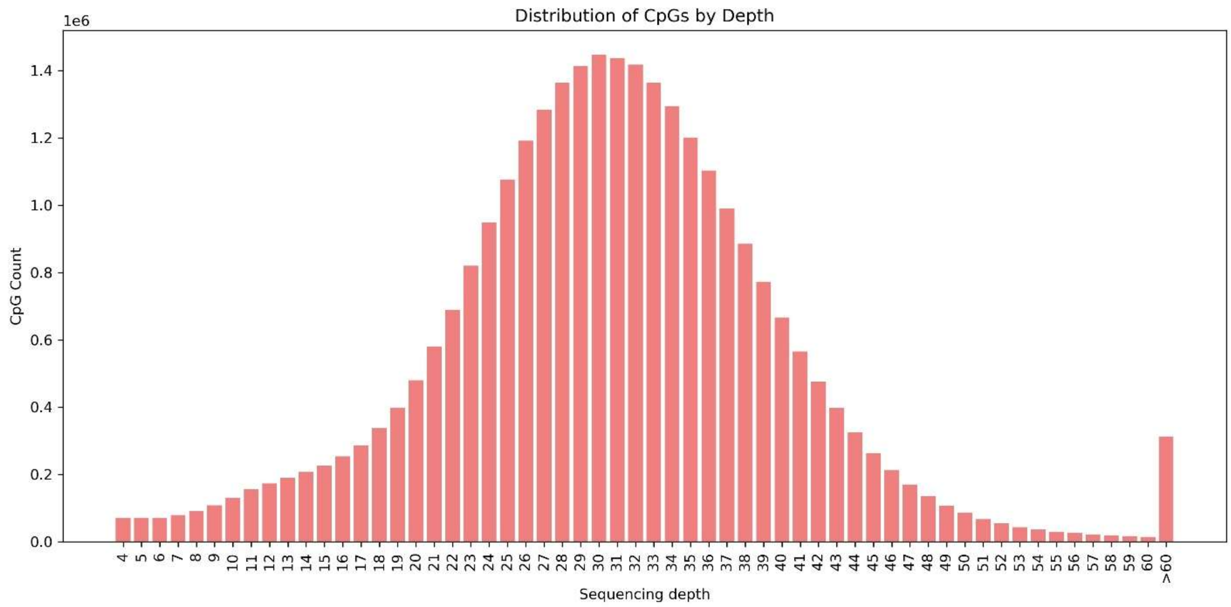

Distribution of CpGs by Depth
1e6
1.4
1.2
1.0
0.8
0.6
0.4
0.2
0.0
CpG Count
4 5 6 7 8 9 10 11 12 13 14 15 16 17 18 19 20 21 22 23 24 25 26 27 28 29 30 31 32 33 34 35 36 37 38 39 40 41 42 43 44 45 46 47 48 49 50 51 52 53 54 55 56 57 58 59 60 >60
Sequencing depth

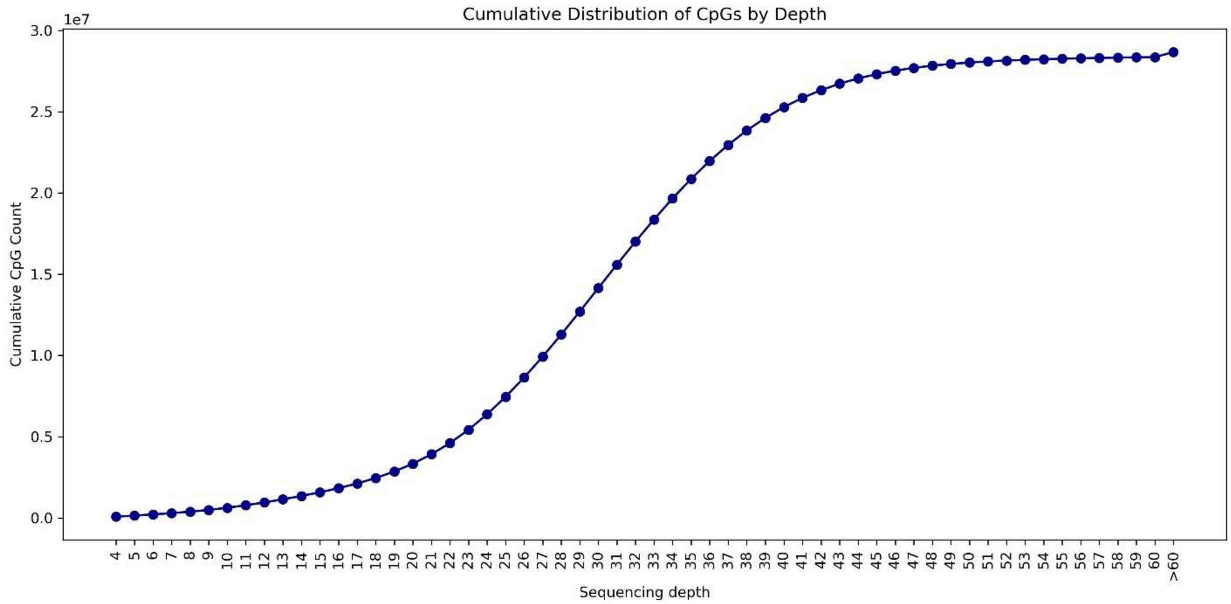

Cumulative Distribution of CpGs by Depth
1e7
3.0
2.5
2.0
1.5
1.0
0.5
0.0
Cumulative CpG Count
4 5 6 7 8 9 10 11 12 13 14 15 16 17 18 19 20 21 22 23 24 25 26 27 28 29 30 31 32 33 34 35 36 37 38 39 40 41 42 43 44 45 46 47 48 49 50 51 52 53 54 55 56 57 58 59 60 >60
Sequencing depth

(B)

Twin A

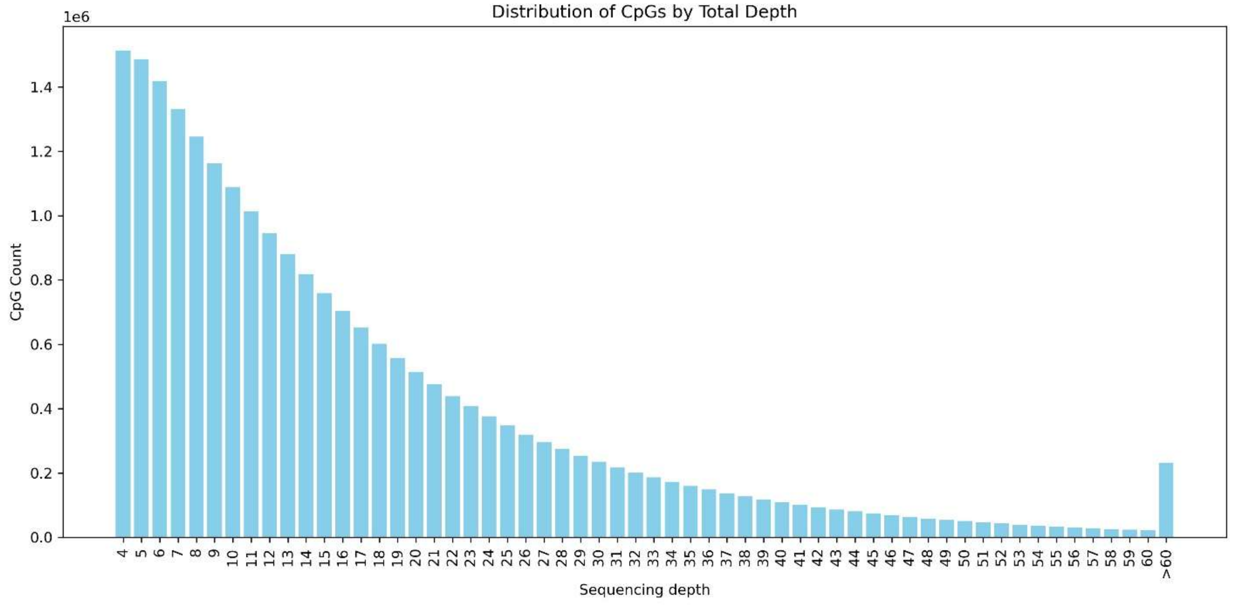
Distribution of CpGs by Total Depth
1e6
CpG Count
0.0
0.2
0.4
0.6
0.8
1.0
1.2
1.4
4 5 6 7 8 9 10 11 12 13 14 15 16 17 18 19 20 21 22 23 24 25 26 27 28 29 30 31 32 33 34 35 36 37 38 39 40 41 42 43 44 45 46 47 48 49 50 51 52 53 54 55 56 57 58 59 60 >60
Sequencing depth

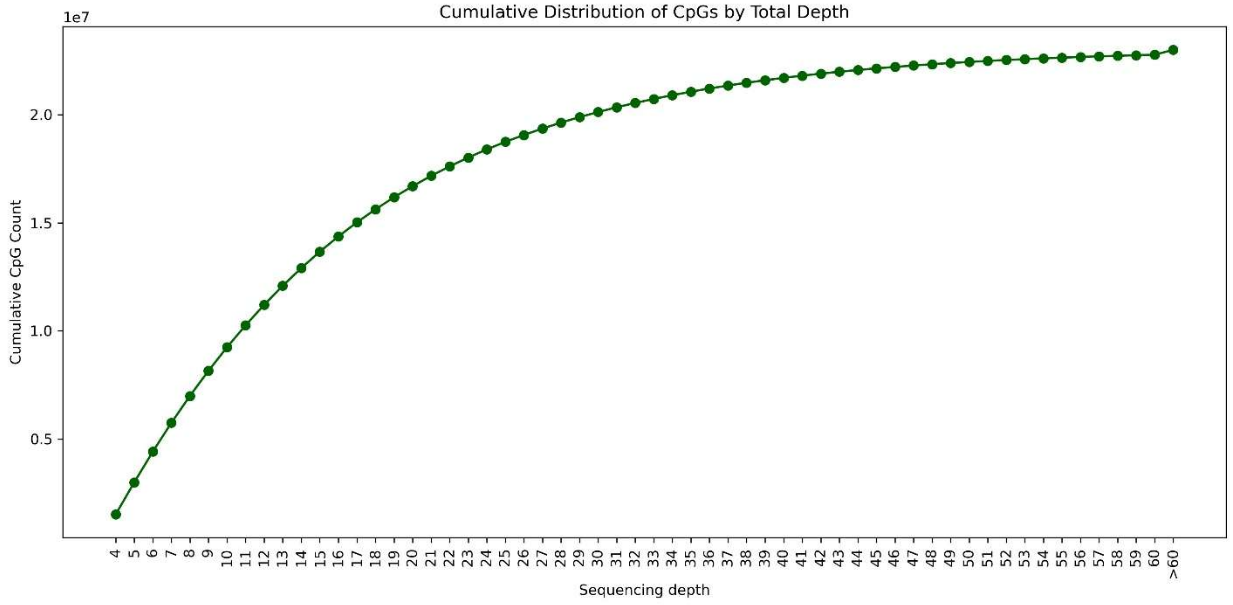
Cumulative Distribution of CpGs by Total Depth
1e7
Cumulative CpG Count
0.5
1.0
1.5
2.0
4 5 6 7 8 9 10 11 12 13 14 15 16 17 18 19 20 21 22 23 24 25 26 27 28 29 30 31 32 33 34 35 36 37 38 39 40 41 42 43 44 45 46 47 48 49 50 51 52 53 54 55 56 57 58 59 60 >60
Sequencing depth

Twin B

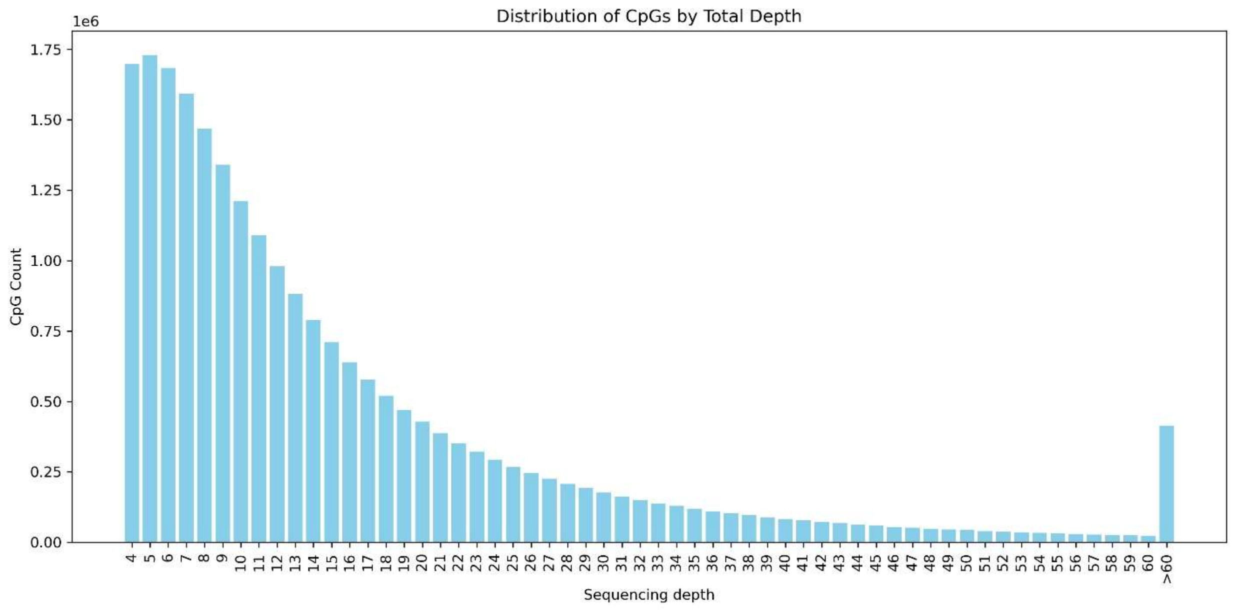

Distribution of CpGs by Total Depth
1e6
CpG Count
Sequencing depth
1.75
1.50
1.25
1.00
0.75
0.50
0.25
0.00
>60

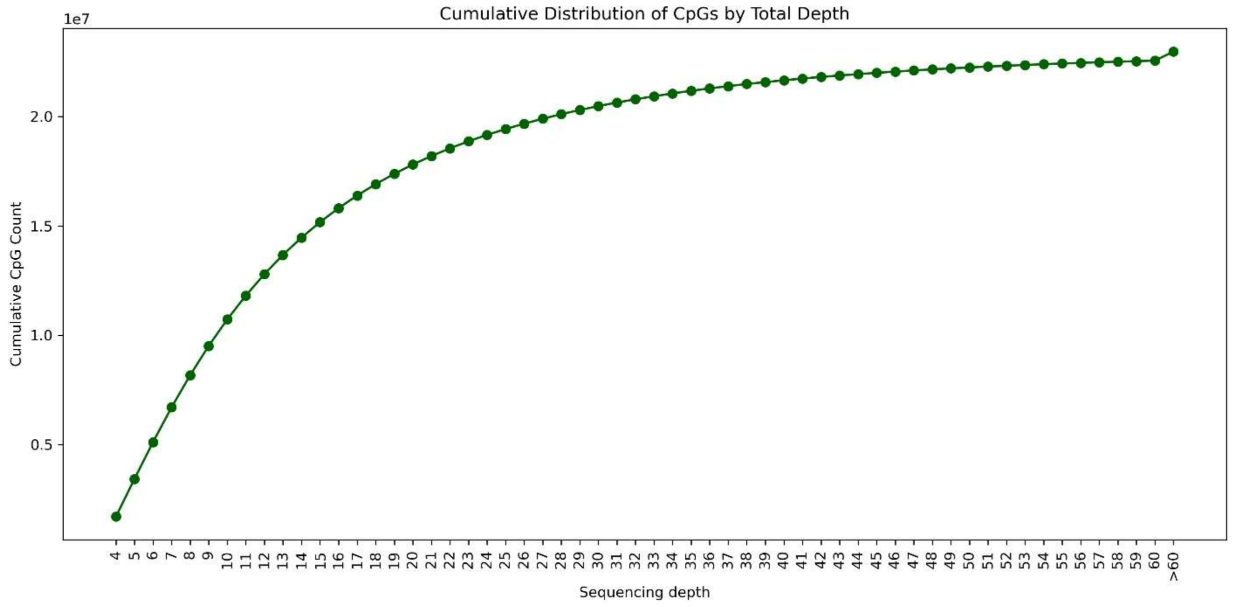

Cumulative Distribution of CpGs by Total Depth
1e7
Cumulative CpG Count
Sequencing depth
2.0
1.5
1.0
0.5
>60

(C)

Twin A

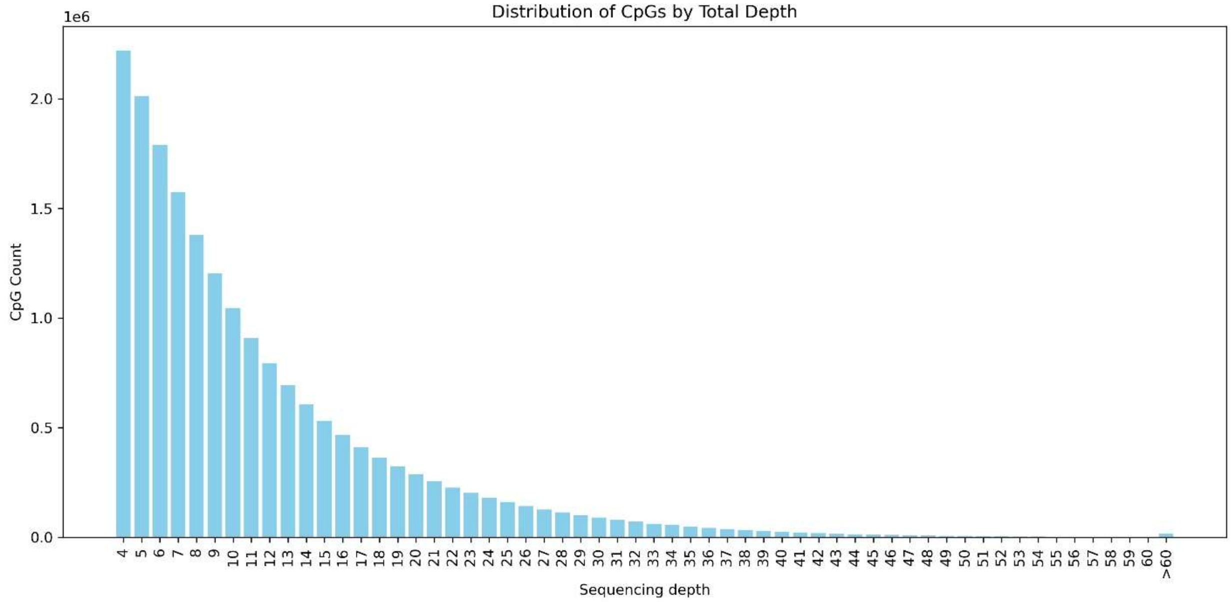

Distribution of CpGs by Total Depth
1e6
CpG Count
0.0
0.5
1.0
1.5
2.0
Sequencing depth
4 5 6 7 8 9 10 11 12 13 14 15 16 17 18 19 20 21 22 23 24 25 26 27 28 29 30 31 32 33 34 35 36 37 38 39 40 41 42 43 44 45 46 47 48 49 50 51 52 53 54 55 56 57 58 59 60 >60

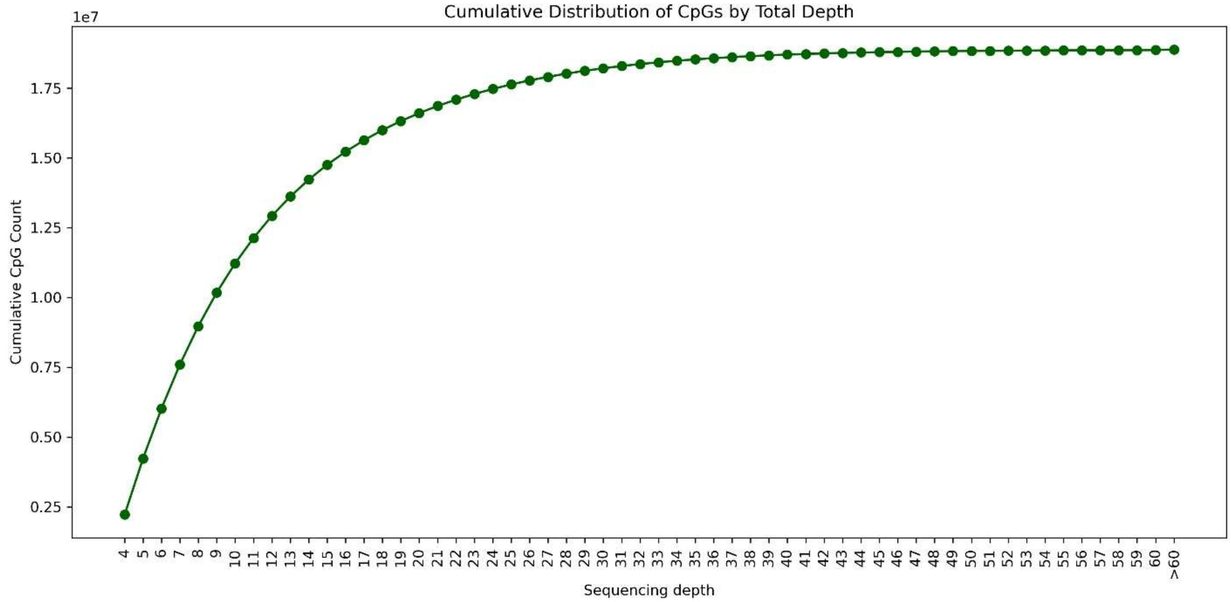

Cumulative Distribution of CpGs by Total Depth
1e7
Cumulative CpG Count
0.25
0.50
0.75
1.00
1.25
1.50
1.75
Sequencing depth
4 5 6 7 8 9 10 11 12 13 14 15 16 17 18 19 20 21 22 23 24 25 26 27 28 29 30 31 32 33 34 35 36 37 38 39 40 41 42 43 44 45 46 47 48 49 50 51 52 53 54 55 56 57 58 59 60 >60

Twin B

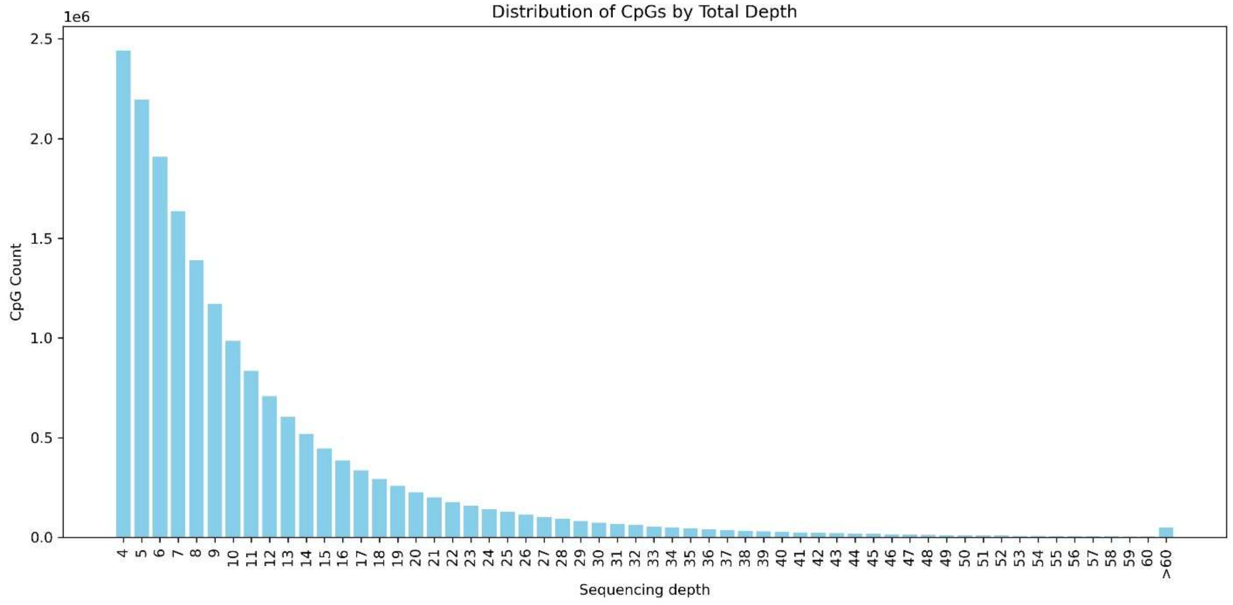

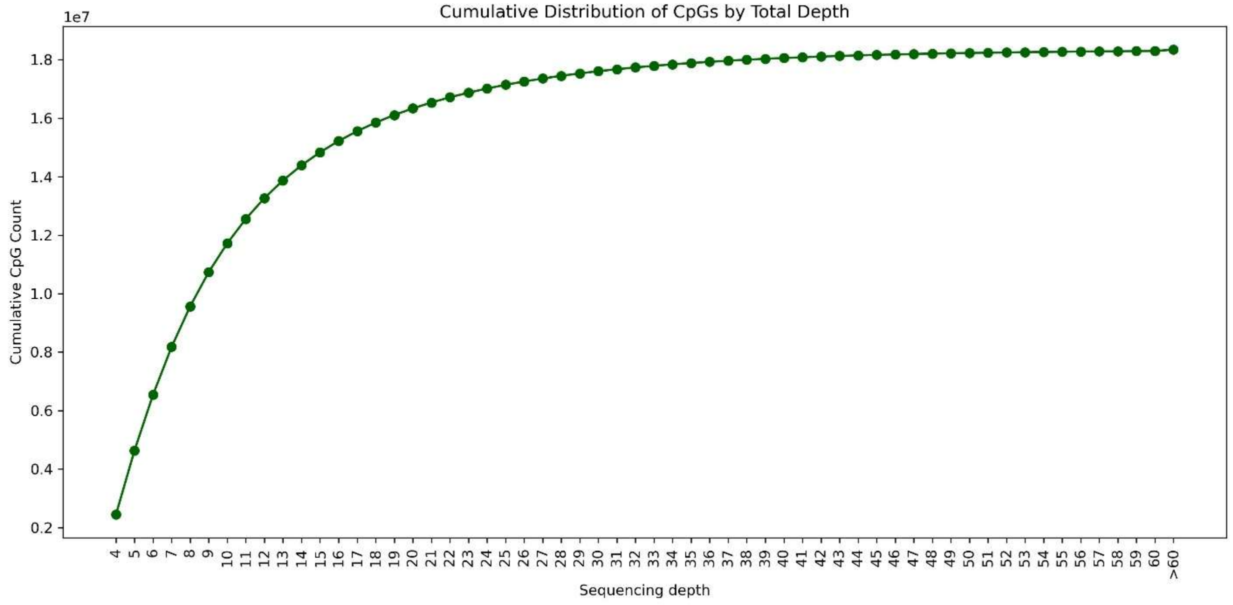

S1 Fig. CpG site detection across increasing coverage thresholds and cumulative coverage. The plots illustrate how the number of detected CpG sites varies with increasing coverage thresholds for (A) HiFi WGS, (B) WGBS (Wg-blimp), (C) WGBS (Bismark)
