# Supplementary material for: A comparison of DNA methylation detection between HiFi sequencing and whole genome bisulfite sequencing in monozygotic twins with Down syndrome
Source: PLoS One. 2025 Aug 5;20(8):e0329593. doi: 10.1371/journal.pone.0329593 (PMC12324119; doi:10.1371/journal.pone.0329593)
Supplement: S2 Fig — Proportions of mCs (defined as ≥50% methylation with ≥4 × read coverage) are shown across sequence-based features: (A) CpG regions (islands, shores, and shelves), (B) CG density categories, and (C) repetitive elements. Data are shown for HiFi WGS, Bismark, overlapping mCs (Overlap), uniquely identified mCs in HiFi WGS (Unique to HiFi WGS), uniquely identified in Bismark (Unique to Bismark), and the difference between the unique sets (Δ unique sites: HiFi WGS vs. Bismark). (PDF) [file pone.0329593.s006.pdf]

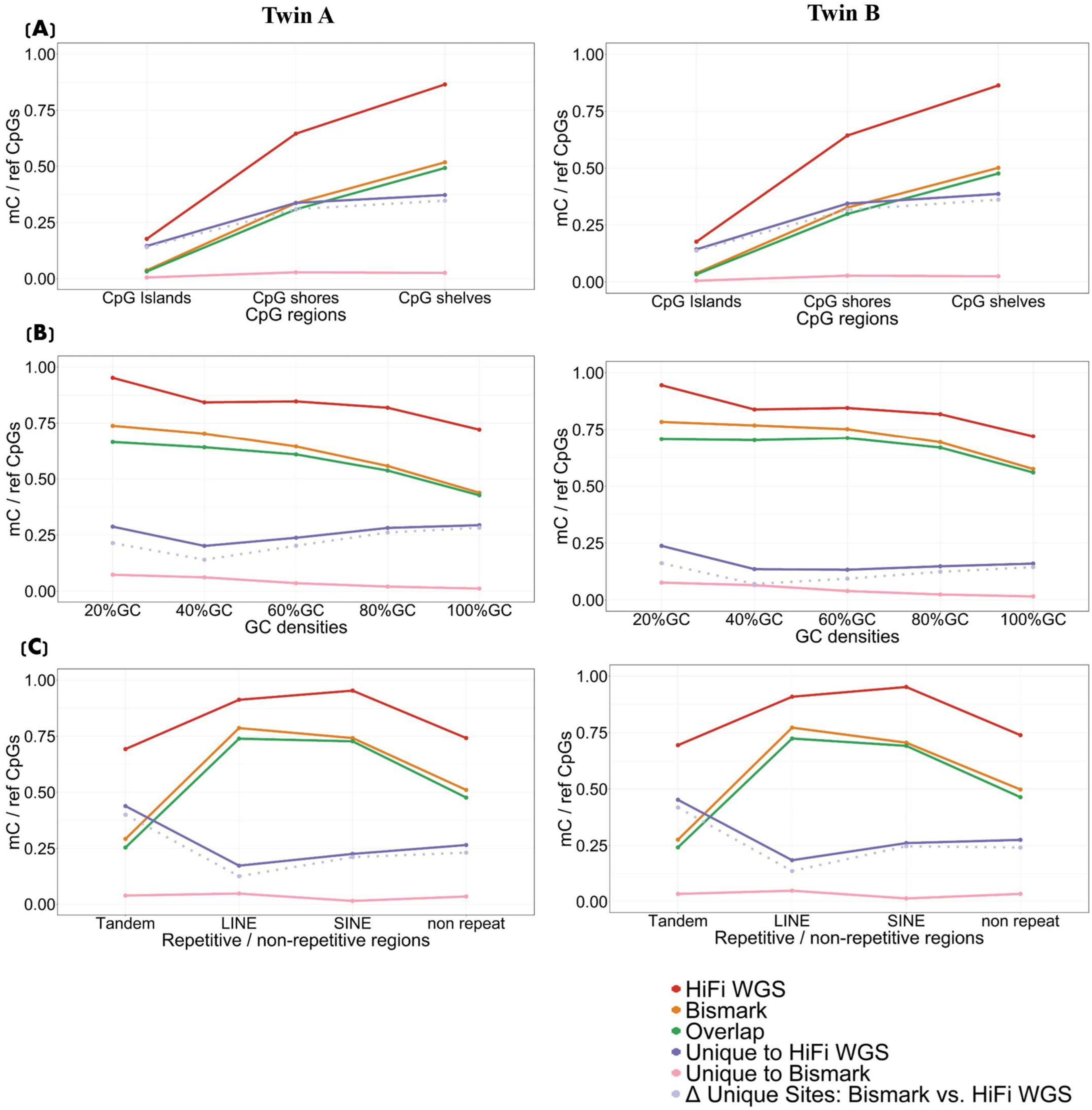

S2 Fig. Distribution of methylated CpGs (≥50% methylation) across primary (sequence-level) genomic contexts in HiFi WGS and WGBS (Bismark). Proportions of methylated CpGs (defined as ≥50% methylation with ≥4× read coverage) are shown across sequence-based features: (A) CpG regions (islands, shores, and shelves), (B) CG density categories, and (C) repetitive elements. Data are shown for HiFi WGS, Bismark, overlapping methylated CpG sites (Overlap), uniquely identified methylated CpGs in HiFi WGS (Unique to HiFi WGS), uniquely identified in Bismark (Unique to Bismark), and the difference between the unique sets (Δ unique sites: HiFi WGS vs. Bismark).
