# Supplementary material for: A comparison of DNA methylation detection between HiFi sequencing and whole genome bisulfite sequencing in monozygotic twins with Down syndrome
Source: PLoS One. 2025 Aug 5;20(8):e0329593. doi: 10.1371/journal.pone.0329593 (PMC12324119; doi:10.1371/journal.pone.0329593)
Supplement: S4 Fig — Proportions of mCs (defined as ≥50% methylation with ≥4 × read coverage) are shown across sequence-based features: (A) CpG regions (islands, shores, and shelves), (B) CG density categories, and (C) repetitive elements. Data are shown for Bismark, wg-blimp, overlapping mCs (Overlap), uniquely identified mCs in Bismark (Unique to Bismark), uniquely identified in wg-blimp (Unique to wg-blimp), and the difference between the unique sets (Δ unique sites: Bismark vs. WGBS). (PDF) [file pone.0329593.s008.pdf]

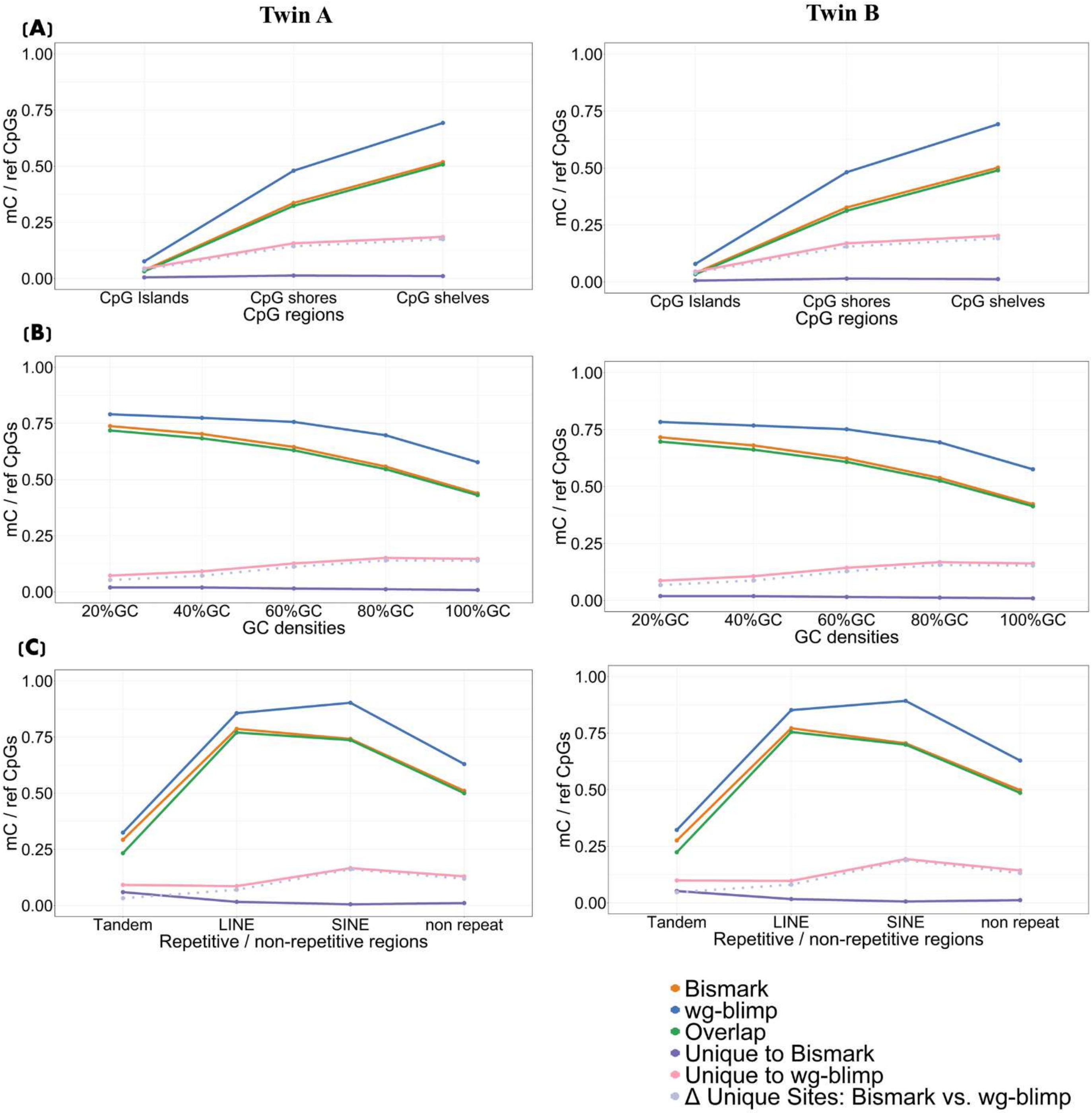

S4 Fig. Distribution of methylated CpGs (≥50% methylation) across primary (sequence-level) genomic contexts in WGBS (Bismark) and WGBS (Wg-blimp). Proportions of methylated CpGs (defined as ≥50% methylation with ≥4× read coverage) are shown across sequence-based features: (A) CpG regions (islands, shores, and shelves), (B) CG density categories, and (C) repetitive elements. Data are shown for Bismark, Wg-blimp, overlapping methylated CpG sites (Overlap), uniquely identified methylated CpGs in Bismark (Unique to Bismark), uniquely identified in Wg-blimp (Unique to Wg-blimp), and the difference between the unique sets (Δ unique sites: Bismark vs. WGBS).
