# Supplementary material for: A comparison of DNA methylation detection between HiFi sequencing and whole genome bisulfite sequencing in monozygotic twins with Down syndrome
Source: PLoS One. 2025 Aug 5;20(8):e0329593. doi: 10.1371/journal.pone.0329593 (PMC12324119; doi:10.1371/journal.pone.0329593)
Supplement: S11 Fig — Methylated CpG proportions (≥80% methylation with ≥4 × read coverage) by: (A) gene-associated regions, (B) regulatory elements (open chromatin and enhancers), and (C) chromosomes. Data are presented for HiFi WGS, Bismark, Overlap, Unique to HiFi WGS, Unique to Bismark, and Δ unique sites. (PDF) [file pone.0329593.s015.pdf]

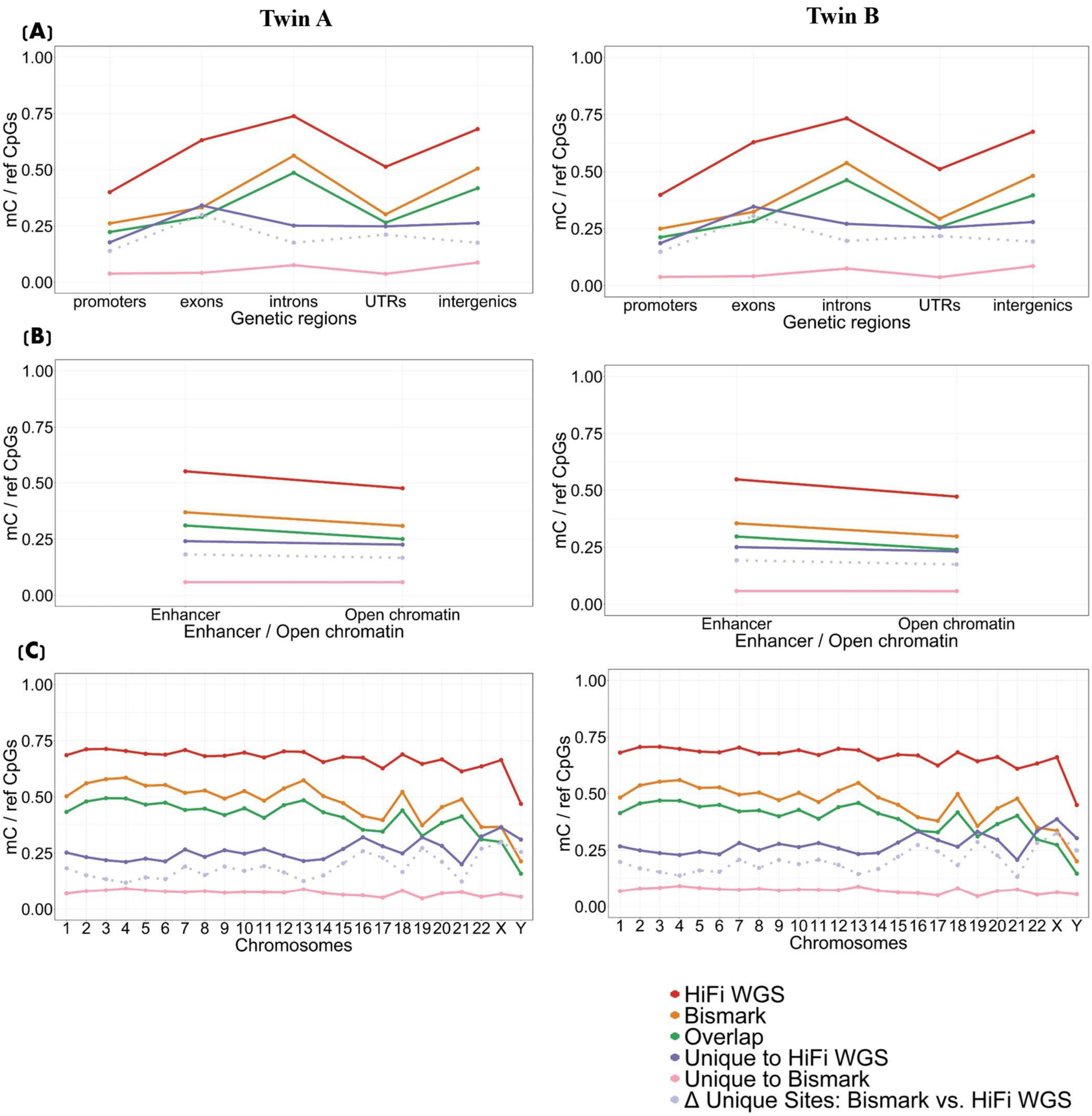

S11 Fig. Distribution of methylated CpGs (≥80% methylation) across secondary (functional level) genomic contexts in HiFi WGS and WGBS (Bismark). Methylated CpG proportions (≥80% methylation with ≥4× read coverage) by: (A) gene-associated regions, (B) regulatory elements (open chromatin and enhancers), and (C) chromosomes. Data are presented for HiFi WGS, Bismark, Overlap, Unique to HiFi WGS, Unique to Bismark, and Δ unique sites.
