# Supplementary material for: A comparison of DNA methylation detection between HiFi sequencing and whole genome bisulfite sequencing in monozygotic twins with Down syndrome
Source: PLoS One. 2025 Aug 5;20(8):e0329593. doi: 10.1371/journal.pone.0329593 (PMC12324119; doi:10.1371/journal.pone.0329593)
Supplement: S12 Fig — Proportion of CpGs from WGBS at corresponding positions to uniquely mC positions detected by HiFi in twins A (WGBS from unique HiFi A) and B (WGBS from unique HiFi B), and CpGs from HiFi at corresponding positions to uniquely mC positions detected by WGBS of twin A (HiFi from unique WGBS A) and B (HiFi from unique WGBS B), considering alternative variants (pink), sequencing depth <4 (blue), and methylation levels < 50 (violet). (PDF) [file pone.0329593.s016.pdf]

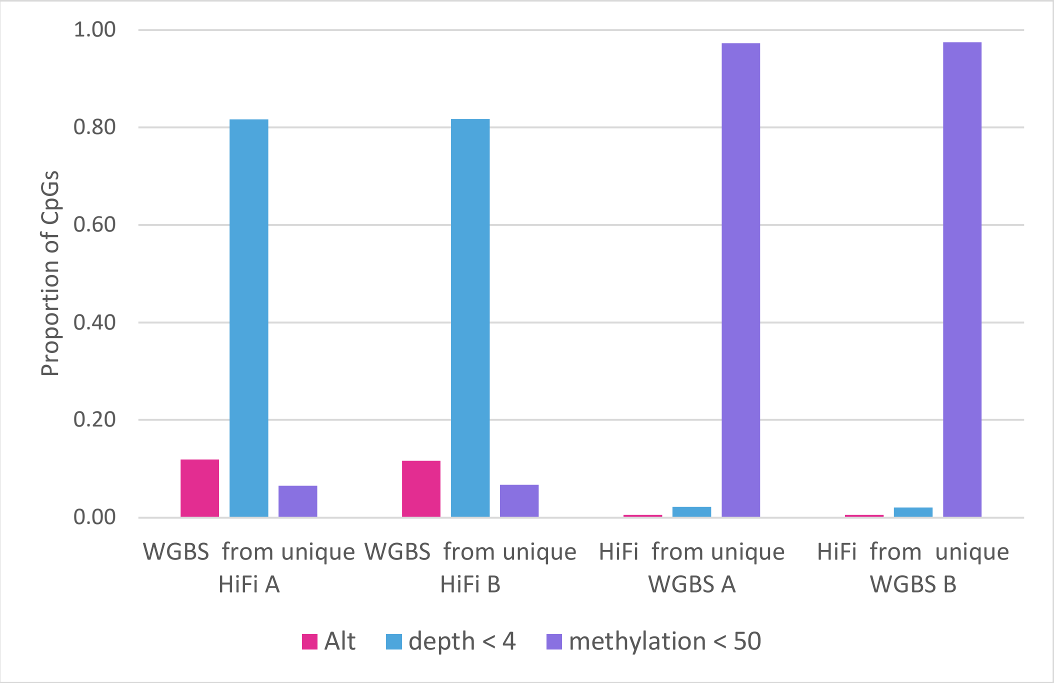

S12 Fig. Examination of mC Position Loss in Each Method. Proportion of CpGs from WGBS at corresponding positions to uniquely mC positions detected by HiFi in twins A (WGBS from unique HiFi A) and B (WGBS from unique HiFi B), and CpGs from HiFi at corresponding positions to uniquely mC positions detected by WGBS of twin A (HiFi from unique WGBS A) and B (HiFi from unique WGBS B), considering alternative variants (pink), sequencing depth <4 (blue), and methylation levels < 50 (violet).
