# Supplementary material for: A comparison of DNA methylation detection between HiFi sequencing and whole genome bisulfite sequencing in monozygotic twins with Down syndrome
Source: PLoS One. 2025 Aug 5;20(8):e0329593. doi: 10.1371/journal.pone.0329593 (PMC12324119; doi:10.1371/journal.pone.0329593)
Supplement: S13 Fig — The proportion of low depth coverage CpGs positions from WGBS at corresponding positions to uniquely mC positions detected by HiFi in twins A (WGBS from unique HiFi A) and twin B (WGBS from unique HiFi B), and CpGs from HIFI at corresponding positions to uniquely mC positions detected by WGBS of twin A (HiFi from unique WGBS A) and twin B (HiFi from unique WGBS B), considering by (A) CpG regions, (B) GC densities, (C) Repeatitive/ non-repeatitive regions, (D) genetic regions and (E) chromosomes. (PDF) [file pone.0329593.s017.pdf]

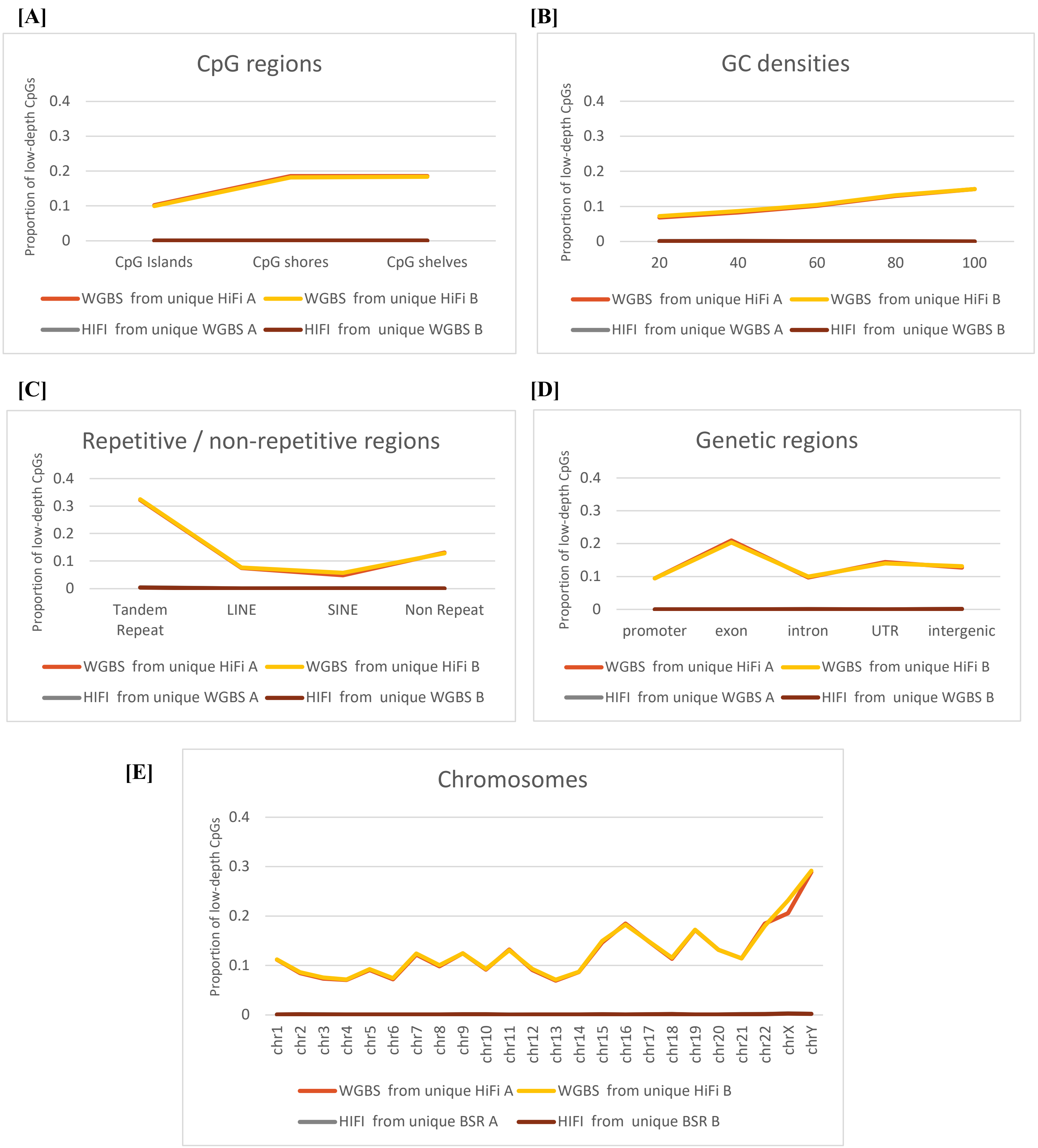

S13 Fig. Proportion of Low-Coverage CpG Sites in Various Genomic Contexts. The proportion of low depth coverage CpGs positions from WGBS at corresponding positions to uniquely mC positions detected by HiFi in twins A (WGBS from unique HiFi A) and twin B (WGBS from unique HiFi B), and CpGs from HIFI at corresponding positions to uniquely mC positions detected by WGBS of twin A (HiFi from unique WGBS A) and twin B (HiFi from unique WGBS B), considering by (A) CpG regions, (B) GC densities, (C) Repeatitive / non-repeatitive regions , (D) genetic regions and (E) chromosomes
