# Supplementary material for: A comparison of DNA methylation detection between HiFi sequencing and whole genome bisulfite sequencing in monozygotic twins with Down syndrome
Source: PLoS One. 2025 Aug 5;20(8):e0329593. doi: 10.1371/journal.pone.0329593 (PMC12324119; doi:10.1371/journal.pone.0329593)
Supplement: S15 Fig — Methylation levels and Pearson correlation between WGBS and HiFi WGS across: (A) gene-associated regions, (B) regulatory regions (open chromatin and enhancers), and (C) chromosomes. (PDF) [file pone.0329593.s019.pdf]

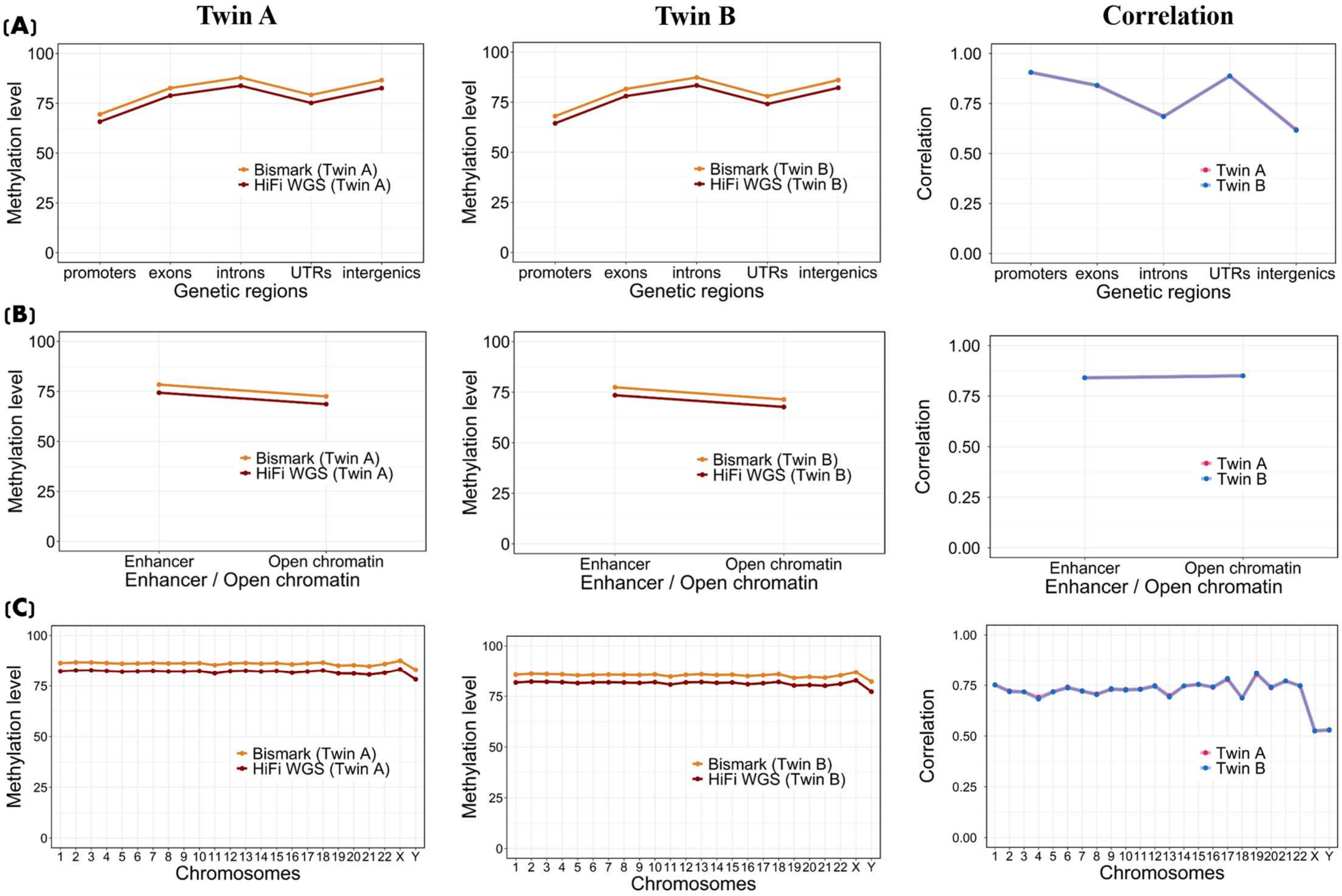

S15 Fig. Methylation levels and correlation across secondary (functional level) genomic contexts in HiFi WGS and WGBS (Bismark). Methylation levels and Pearson correlation between WGBS and HiFi WGS across: (A) gene-associated regions, (B) regulatory regions (open chromatin and enhancers), and (C) chromosomes.
