# Supplementary material for: A comparison of DNA methylation detection between HiFi sequencing and whole genome bisulfite sequencing in monozygotic twins with Down syndrome
Source: PLoS One. 2025 Aug 5;20(8):e0329593. doi: 10.1371/journal.pone.0329593 (PMC12324119; doi:10.1371/journal.pone.0329593)
Supplement: S16 Fig — Methylation levels (methylation probabilities) and Pearson correlation between WGBS and HiFi WGS across: (A) CpG regions (CpG islands, shores, and shelves), (B) CG density categories, and (C) repetitive elements. (PDF) [file pone.0329593.s020.pdf]

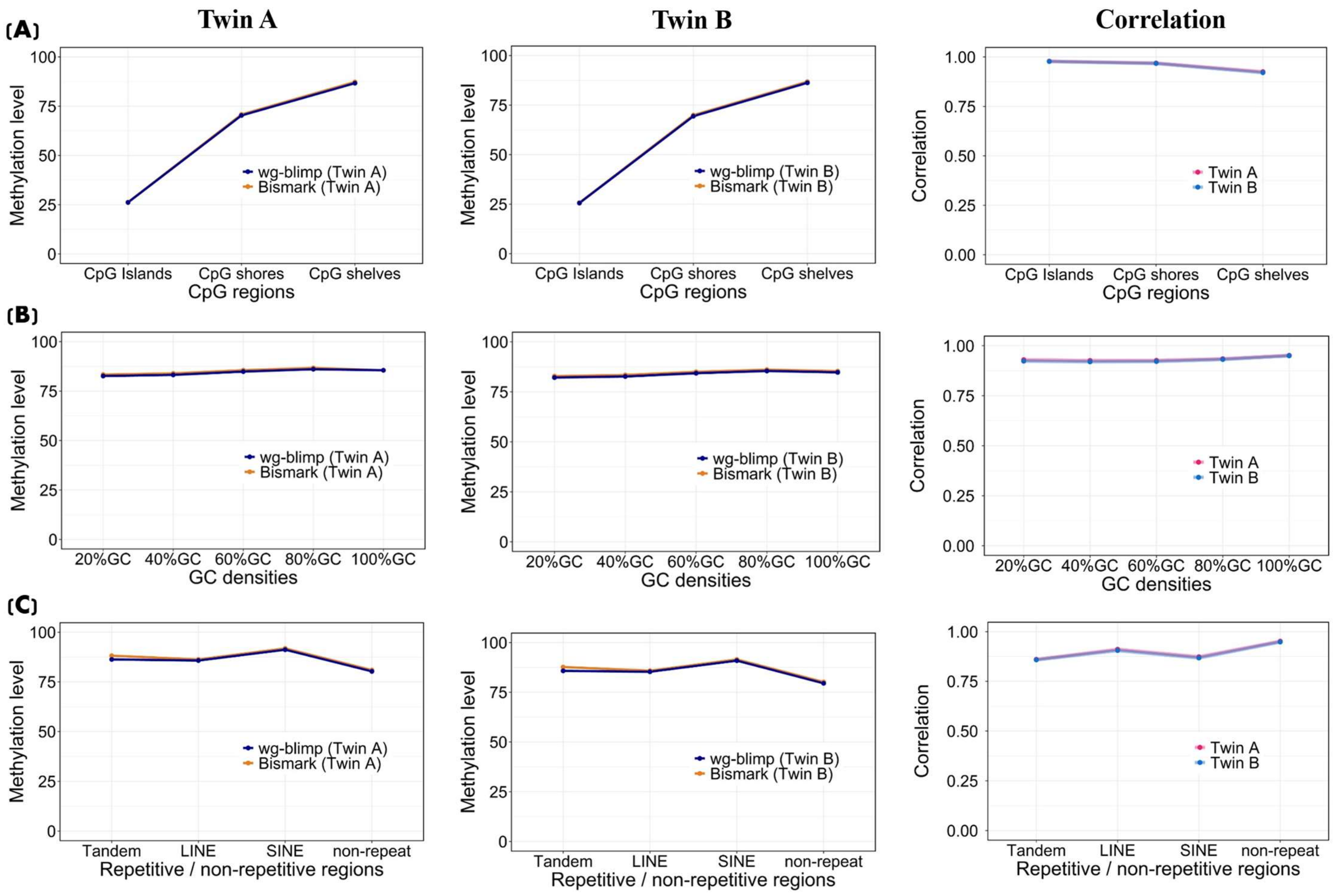

S16 Fig. Methylation levels and correlation across primary (sequence-level) genomic contexts in WGBS (Bismark) and WGBS (Wg-blimp). Methylation levels (methylation probabilities) and Pearson correlation between WGBS and HiFi WGS across: (A) CpG regions (CpG islands, shores, and shelves), (B) CG density categories, and (C) repetitive elements.
