# Supplementary material for: A comparison of DNA methylation detection between HiFi sequencing and whole genome bisulfite sequencing in monozygotic twins with Down syndrome
Source: PLoS One. 2025 Aug 5;20(8):e0329593. doi: 10.1371/journal.pone.0329593 (PMC12324119; doi:10.1371/journal.pone.0329593)
Supplement: S17 Fig — Methylation levels and Pearson correlation between WGBS and HiFi WGS across: (A) gene-associated regions, (B) regulatory regions (open chromatin and enhancers), and (C) chromosomes. (PDF) [file pone.0329593.s021.pdf]

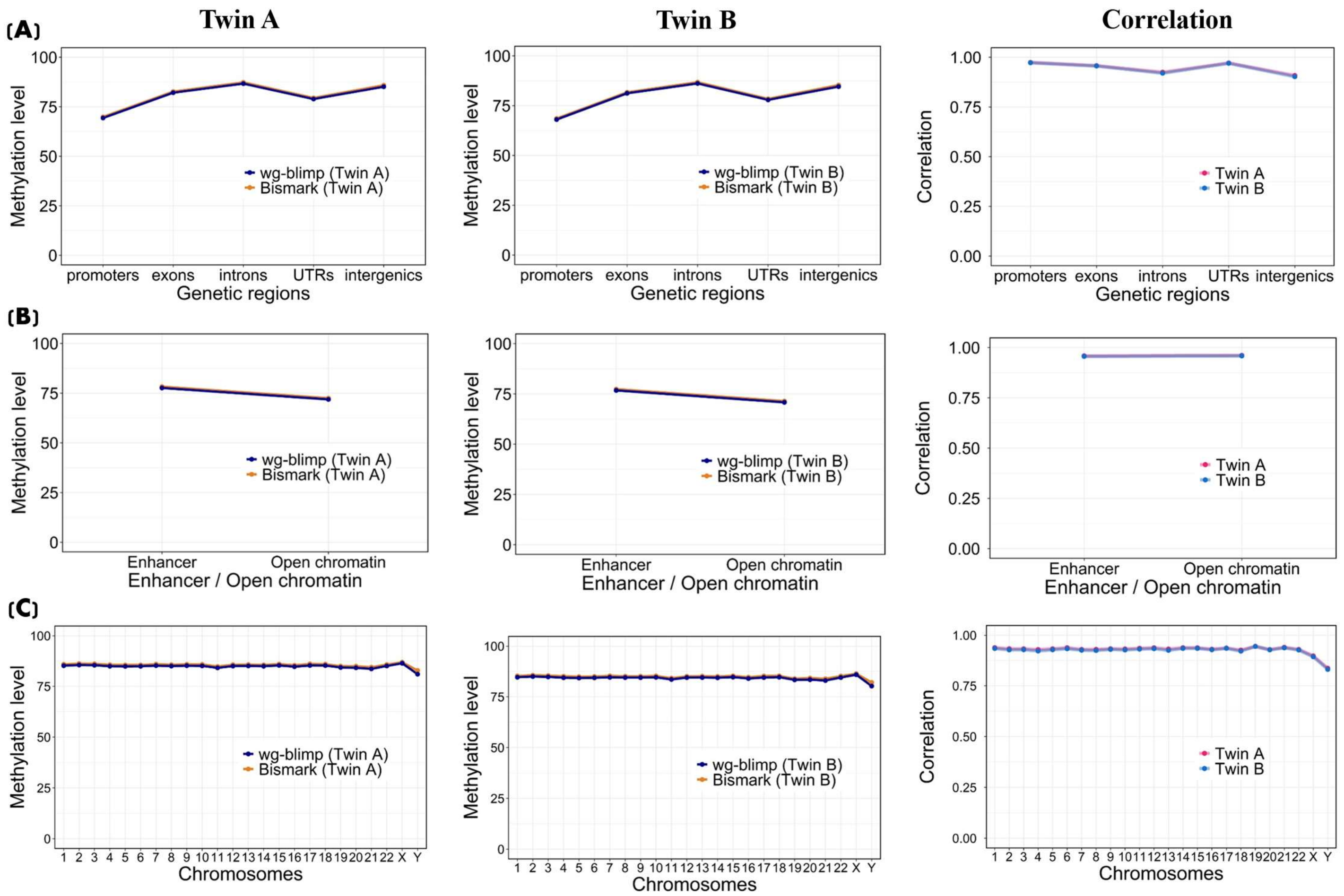

S17 Fig. Methylation levels and correlation across secondary (functional level) genomic contexts in WGBS (Bismark) and WGBS (Wg-blimp). Methylation levels and Pearson correlation between WGBS and HiFi WGS across: (A) gene-associated regions, (B) regulatory regions (open chromatin and enhancers), and (C) chromosomes.
