# Supplementary material for: A comparison of DNA methylation detection between HiFi sequencing and whole genome bisulfite sequencing in monozygotic twins with Down syndrome
Source: PLoS One. 2025 Aug 5;20(8):e0329593. doi: 10.1371/journal.pone.0329593 (PMC12324119; doi:10.1371/journal.pone.0329593)
Supplement: S18 Fig — Average methylation levels are plotted relative to gene structures, including 2 kb upstream of the transcription start site (TSS), the gene body (scaled to uniform length), and 2 kb downstream of the transcription end site (TES). The plot compares methylation patterns in promoter, gene body, and downstream regions across platforms (HiFi WGS, Bismark, and wg-blimp (MethylDackel). (PDF) [file pone.0329593.s022.pdf]

Twin A

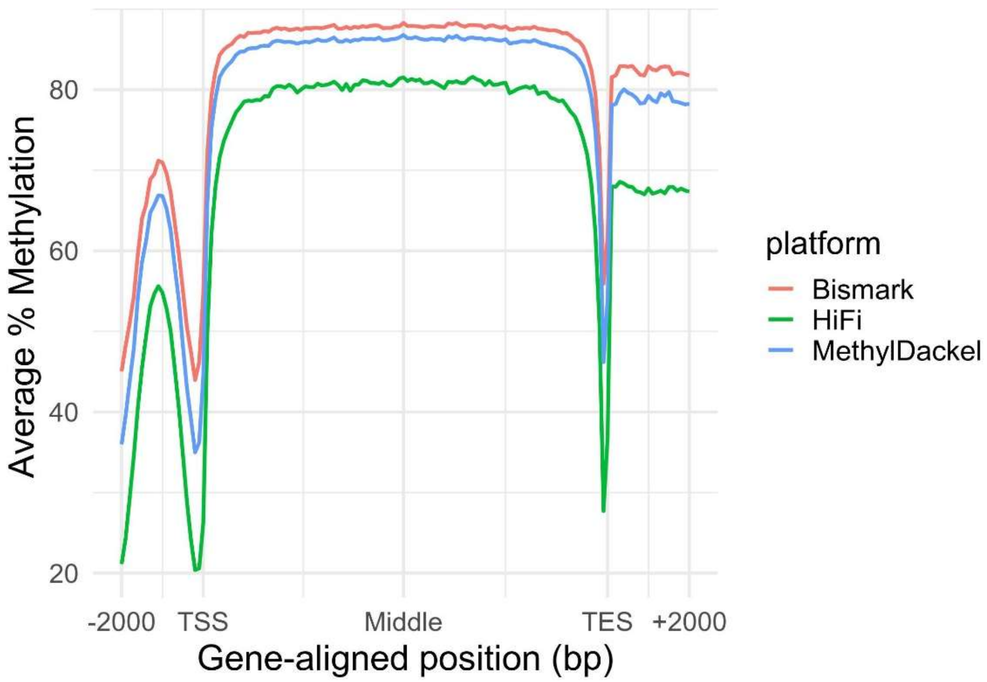

Twin B

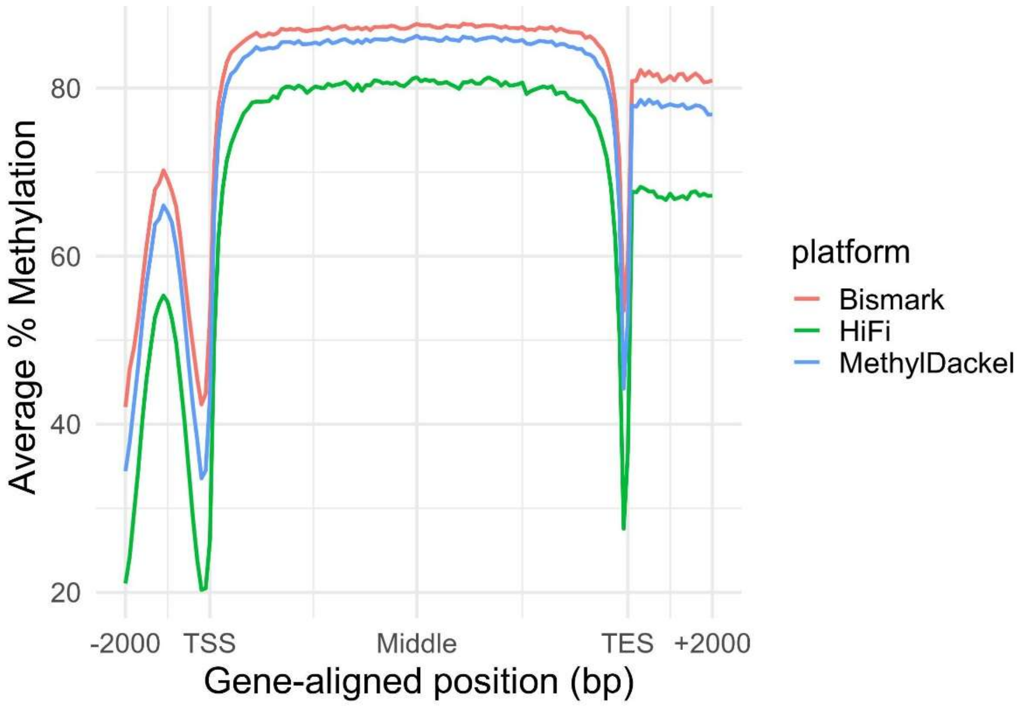

S18 Fig. Methylation signal distribution relative to gene structure. Average methylation levels are plotted relative to gene structures, including 2 kb upstream of the transcription start site (TSS), the gene body (scaled to uniform length), and 2 kb downstream of the transcription end site (TES). The plot compares methylation patterns in promoter, gene body, and downstream regions across platforms (HiFi WGS, Bismark, and Wg-blimp (MethylDackel).
