# Supplementary material for: A comparison of DNA methylation detection between HiFi sequencing and whole genome bisulfite sequencing in monozygotic twins with Down syndrome
Source: PLoS One. 2025 Aug 5;20(8):e0329593. doi: 10.1371/journal.pone.0329593 (PMC12324119; doi:10.1371/journal.pone.0329593)
Supplement: S19 Fig — Comparisons are shown across: (A) CpG-related regions (islands, shores, and shelves), (B) CG density categories, (C) repetitive elements, (D) gene-associated regions, and (E) regulatory regions (open chromatin and enhancers). (PDF) [file pone.0329593.s023.pdf]

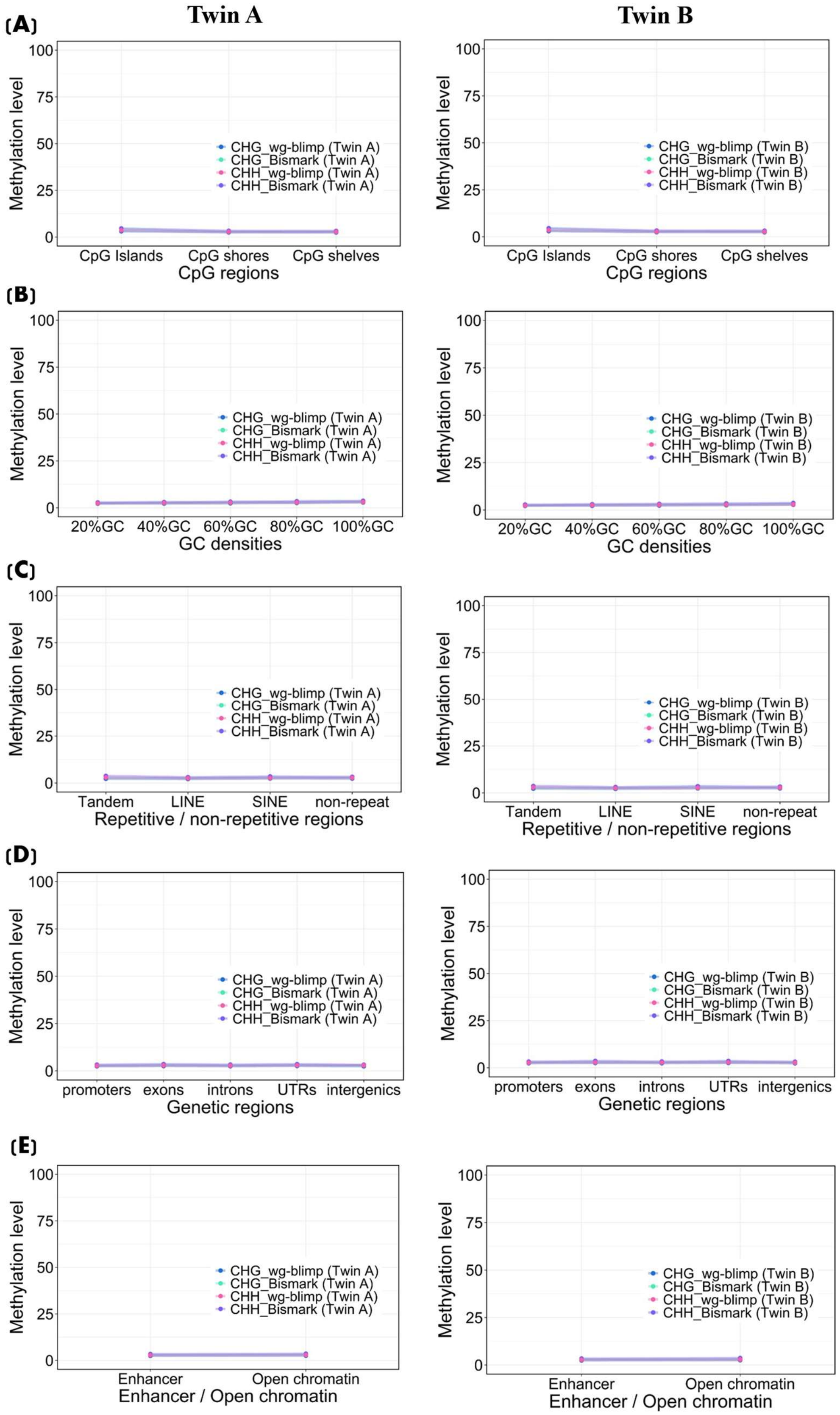

S19 Fig. Methylation levels of non-CpG sites (CHG and CHH) across genomic contexts in WGBS (wg-blimp), and WGBS (Bismark). Comparisons are shown across: (A) CpG-related regions (islands, shores, and shelves), (B) CG density categories, (C) repetitive elements, (D) gene-associated regions, and (E) regulatory regions (open chromatin and enhancers).
