# Supplementary material for: A comparison of DNA methylation detection between HiFi sequencing and whole genome bisulfite sequencing in monozygotic twins with Down syndrome
Source: PLoS One. 2025 Aug 5;20(8):e0329593. doi: 10.1371/journal.pone.0329593 (PMC12324119; doi:10.1371/journal.pone.0329593)
Supplement: S20 Fig — Scatter plots (2D binned heatmaps) with linear regression lines illustrate methylation level concordance between WGBS and HiFi for CpG sites across: (A) CpG regions (islands, shores, and shelves), CG density categories, repetitive elements, (B) gene-associated regions, and regulatory regions (open chromatin and enhancers). (PDF) [file pone.0329593.s024.pdf]

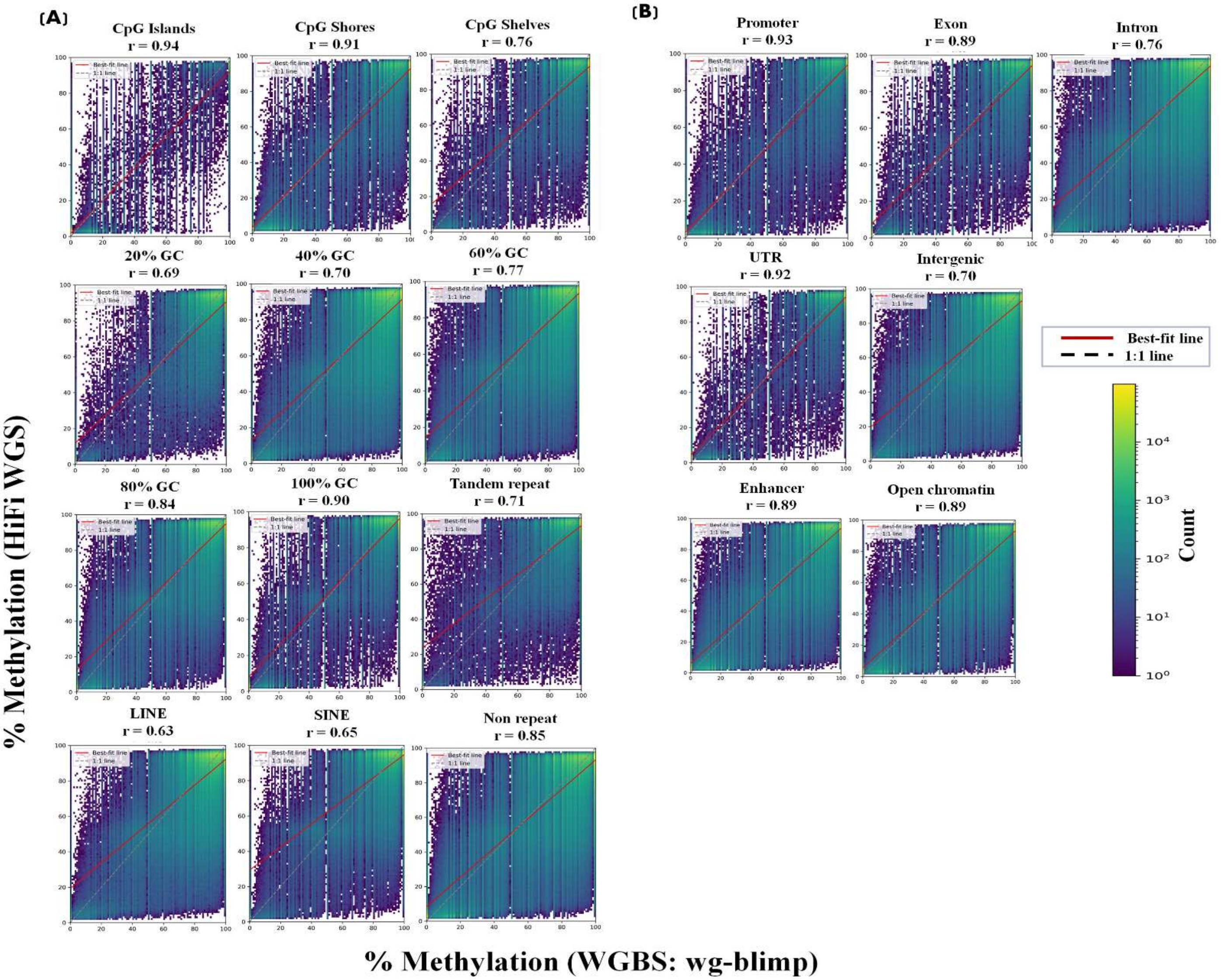

S20 Fig. Two-dimensional heatmaps with best-fit lines showing methylation concordance between WGBS (Wg-blimp) and HiFi WGS across genomic contexts (Twin B). Scatter plots (2D binned heatmaps) with linear regression lines illustrate methylation level concordance between WGBS and HiFi for CpG sites across: (A) CpG regions (islands, shores, and shelves), CG density categories, repetitive elements, (B) gene-associated regions, and regulatory regions (open chromatin and enhancers).
