# Supplementary material for: A comparison of DNA methylation detection between HiFi sequencing and whole genome bisulfite sequencing in monozygotic twins with Down syndrome
Source: PLoS One. 2025 Aug 5;20(8):e0329593. doi: 10.1371/journal.pone.0329593 (PMC12324119; doi:10.1371/journal.pone.0329593)
Supplement: S21 Fig — Proportions of genetic regions (promoters, exons, introns, UTRs, intergenic regions) categorized by GC density levels (20, 40, 60, 80, and 100%). (PDF) [file pone.0329593.s025.pdf]

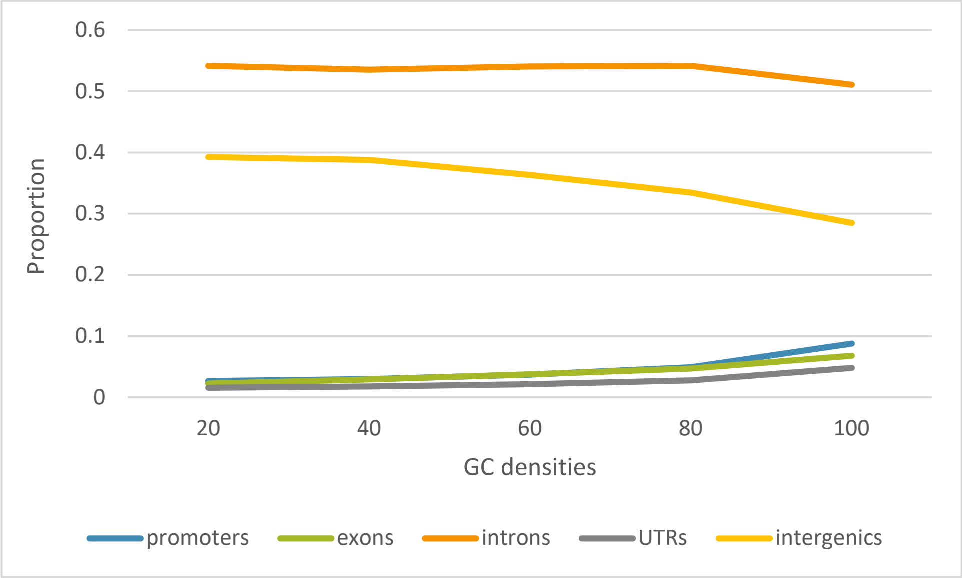

S21 Fig. Distribution of genetic regions across different GC density bins. Proportions of genetic regions (promoters, exons, introns, UTRs, intergenic regions) categorized by GC density levels (20, 40, 60, 80, and 100%).
