# Supplementary material for: A comparison of DNA methylation detection between HiFi sequencing and whole genome bisulfite sequencing in monozygotic twins with Down syndrome
Source: PLoS One. 2025 Aug 5;20(8):e0329593. doi: 10.1371/journal.pone.0329593 (PMC12324119; doi:10.1371/journal.pone.0329593)
Supplement: S22 Fig — Proportion of (A) CpG regions, (B) GC density categories, and (C) genetic regions across each chromosome based on overlapping CpG positions identified by WGBS (wg-blimp) and HiFi WGS in the twin samples. (PDF) [file pone.0329593.s026.pdf]

[A]

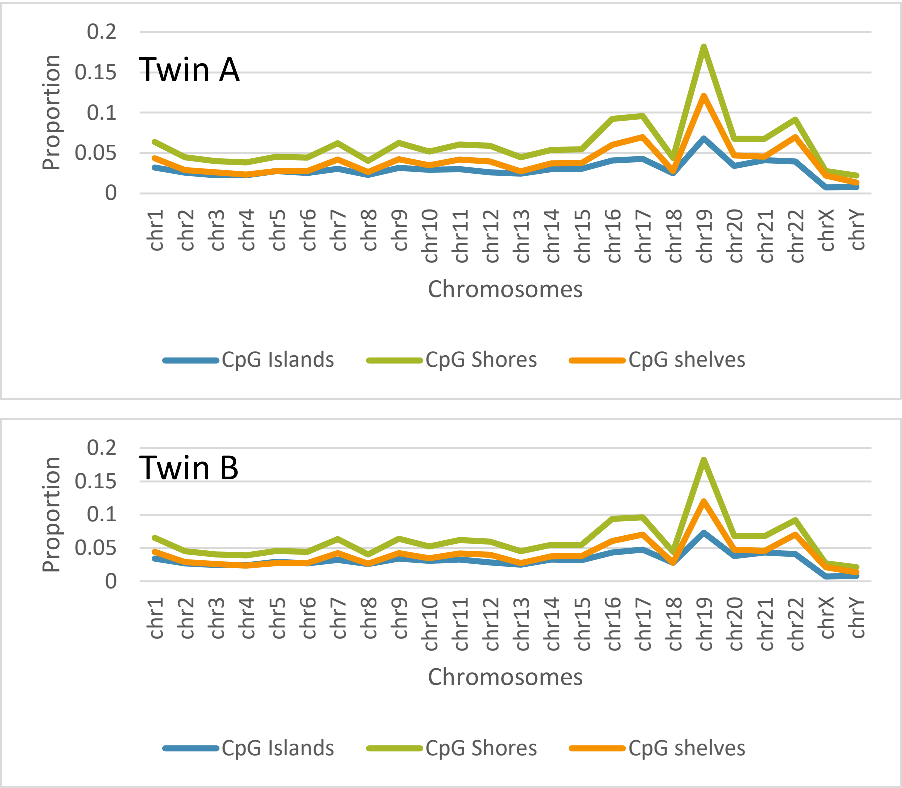
Twin A
Proportion
0.2
0.15
0.1
0.05
0
chr1 chr2 chr3 chr4 chr5 chr6 chr7 chr8 chr9 chr10 chr11 chr12 chr13 chr14 chr15 chr16 chr17 chr18 chr19 chr20 chr21 chr22 chrX chrY
Chromosomes
CpG Islands
CpG Shores
CpG shelves
Twin B
Proportion
0.2
0.15
0.1
0.05
0
chr1 chr2 chr3 chr4 chr5 chr6 chr7 chr8 chr9 chr10 chr11 chr12 chr13 chr14 chr15 chr16 chr17 chr18 chr19 chr20 chr21 chr22 chrX chrY
Chromosomes
CpG Islands
CpG Shores
CpG shelves

[B]

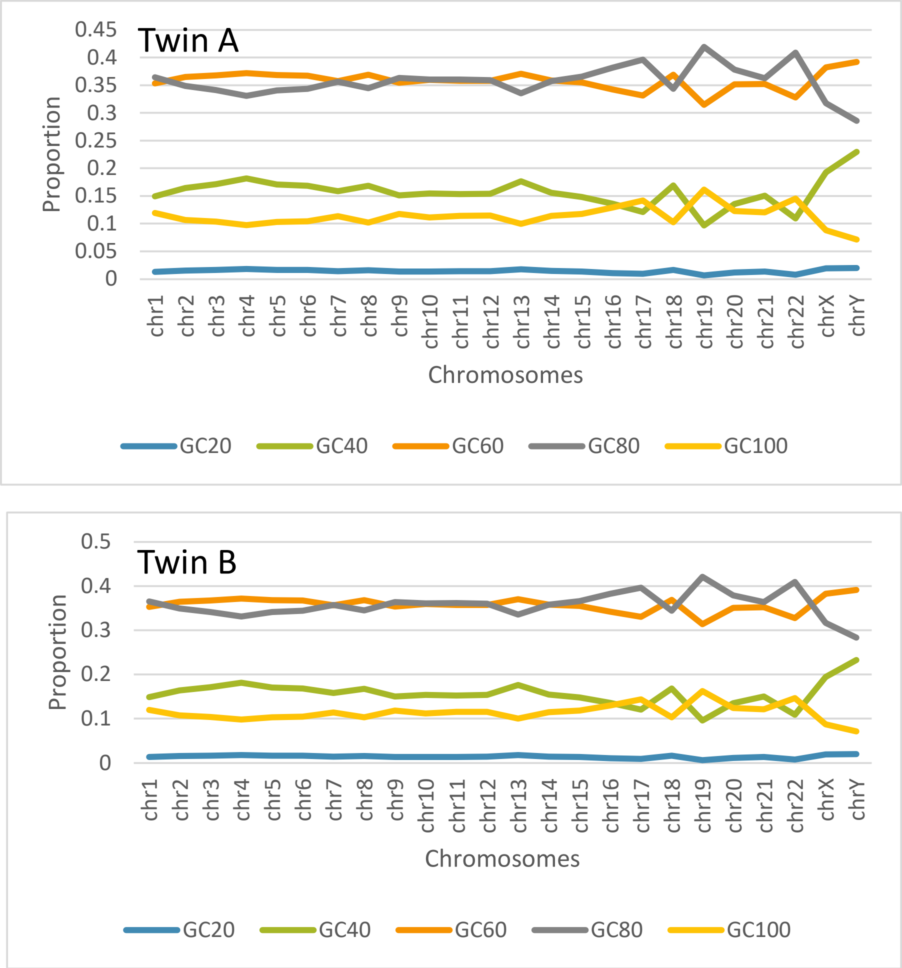
Twin A
Proportion
0.45
0.4
0.35
0.3
0.25
0.2
0.15
0.1
0.05
0
chr1 chr2 chr3 chr4 chr5 chr6 chr7 chr8 chr9 chr10 chr11 chr12 chr13 chr14 chr15 chr16 chr17 chr18 chr19 chr20 chr21 chr22 chrX chrY
Chromosomes
GC20
GC40
GC60
GC80
GC100
Twin B
Proportion
0.5
0.4
0.3
0.2
0.1
0
chr1 chr2 chr3 chr4 chr5 chr6 chr7 chr8 chr9 chr10 chr11 chr12 chr13 chr14 chr15 chr16 chr17 chr18 chr19 chr20 chr21 chr22 chrX chrY
Chromosomes
GC20
GC40
GC60
GC80
GC100

[C]

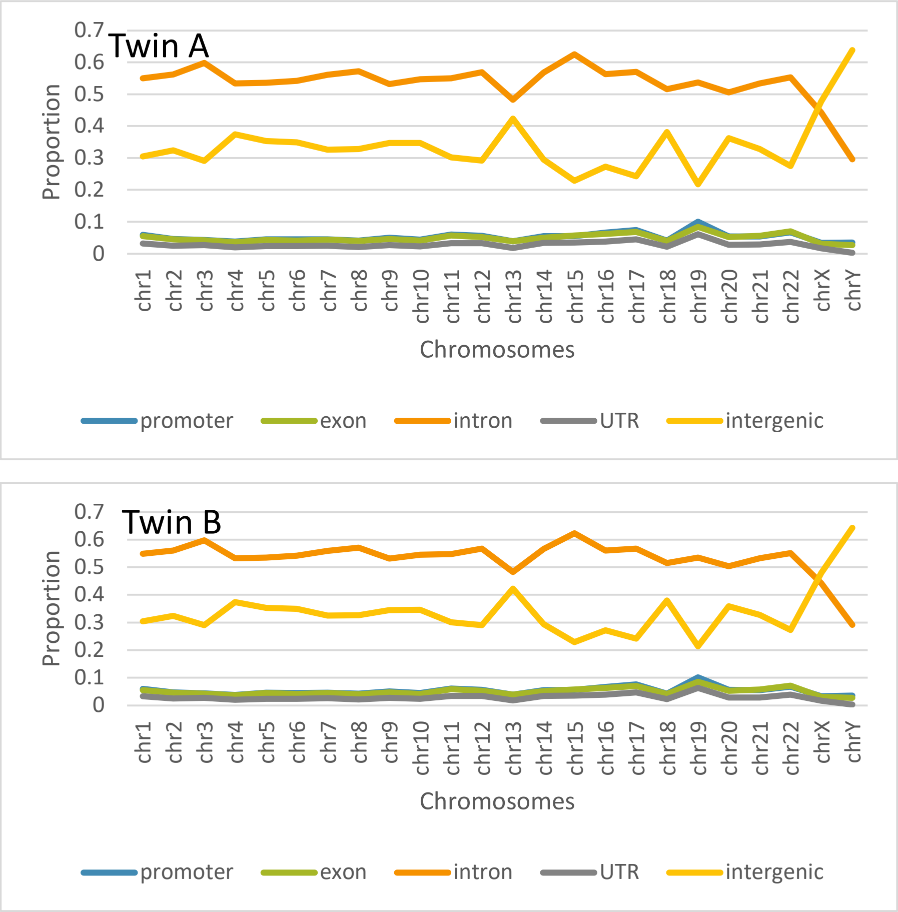

S22 Fig. Genomic context distribution across chromosomes. Proportion of (A) CpG regions, (B) GC density categories, and (C) genetic regions across each chromosome based on overlapping CpG positions identified by WGBS (wg-blimp) and HiFi WGS in the twin samples.
