# Supplementary material for: A comparison of DNA methylation detection between HiFi sequencing and whole genome bisulfite sequencing in monozygotic twins with Down syndrome
Source: PLoS One. 2025 Aug 5;20(8):e0329593. doi: 10.1371/journal.pone.0329593 (PMC12324119; doi:10.1371/journal.pone.0329593)
Supplement: S23 Fig — Methylated and unmethylated counts were randomly downsampled at each CpG site to the lower read depth between platforms, ensuring matched coverage for fair comparison. Pearson correlation coefficients (r) of methylation levels were calculated over 1,000 subsampling iterations. (A) Boxplots of correlation coefficients across genomic contexts. (B) Boxplots of correlation coefficients stratified by depth bins. (PDF) [file pone.0329593.s027.pdf]

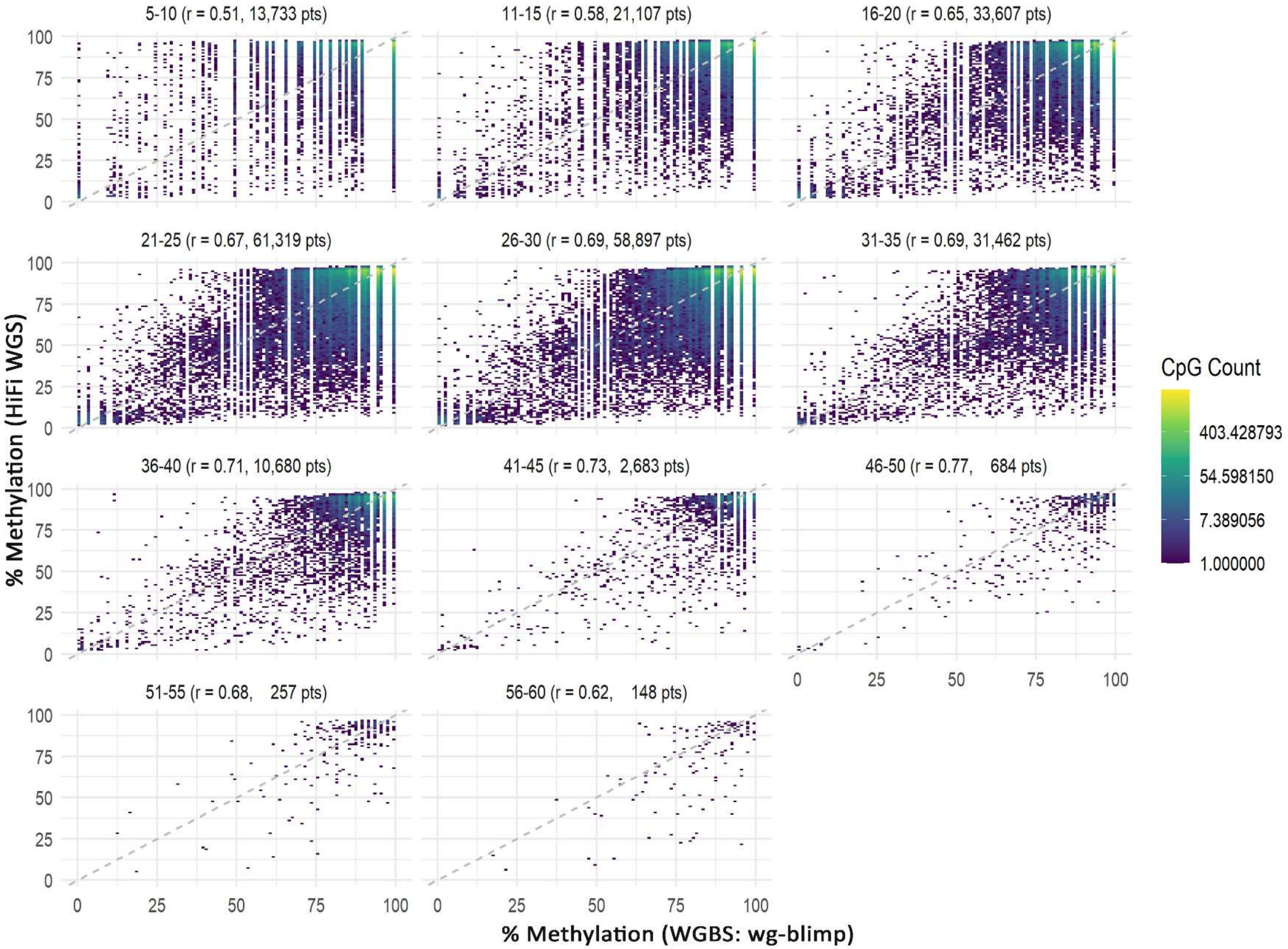

S23 Fig. Methylation concordance between HiFi WGS and WGBS after depth-matched subsampling (Twin B). Methylated and unmethylated counts were randomly downsampled at each CpG site to the lower read depth between platforms, ensuring matched coverage for fair comparison. Pearson correlation coefficients (r) of methylation levels were calculated over 1,000 subsampling iterations. (A) Boxplots of correlation coefficients across genomic contexts. (B) Boxplots of correlation coefficients stratified by depth bins.
