# Supplementary material for: A comparison of DNA methylation detection between HiFi sequencing and whole genome bisulfite sequencing in monozygotic twins with Down syndrome
Source: PLoS One. 2025 Aug 5;20(8):e0329593. doi: 10.1371/journal.pone.0329593 (PMC12324119; doi:10.1371/journal.pone.0329593)
Supplement: S24 Fig — Downsampling was performed as described in Fig 8. CpG sites were stratified by coverage depth bins. Boxplots show the distribution of Pearson correlation coefficients (r) across 1,000 iterations for each bin. (A) Twin A. (B) Twin B. (PDF) [file pone.0329593.s028.pdf]

[A] Twin A

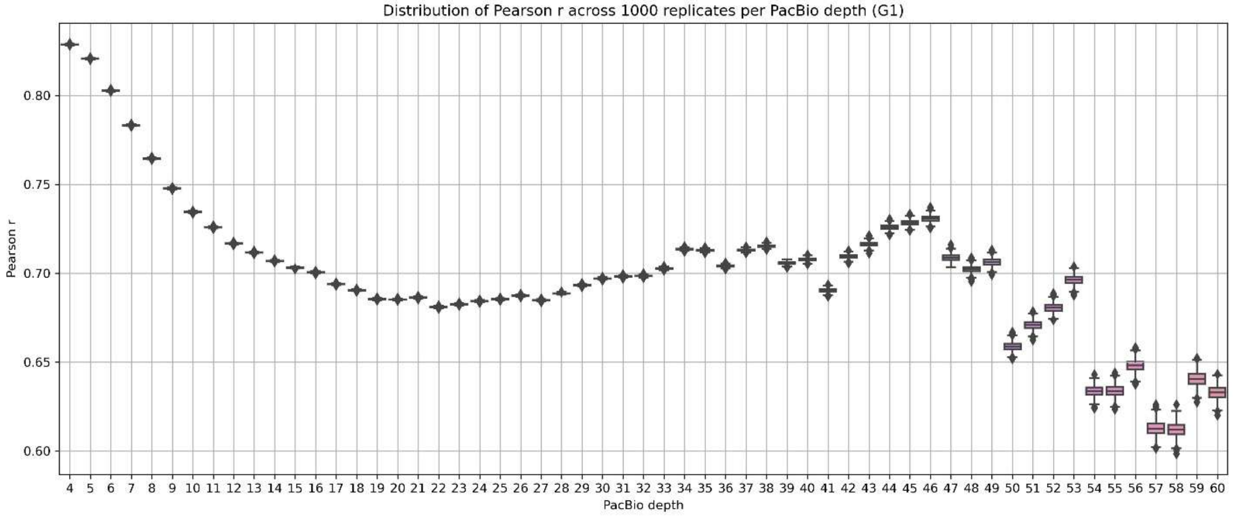

[B] Twin B

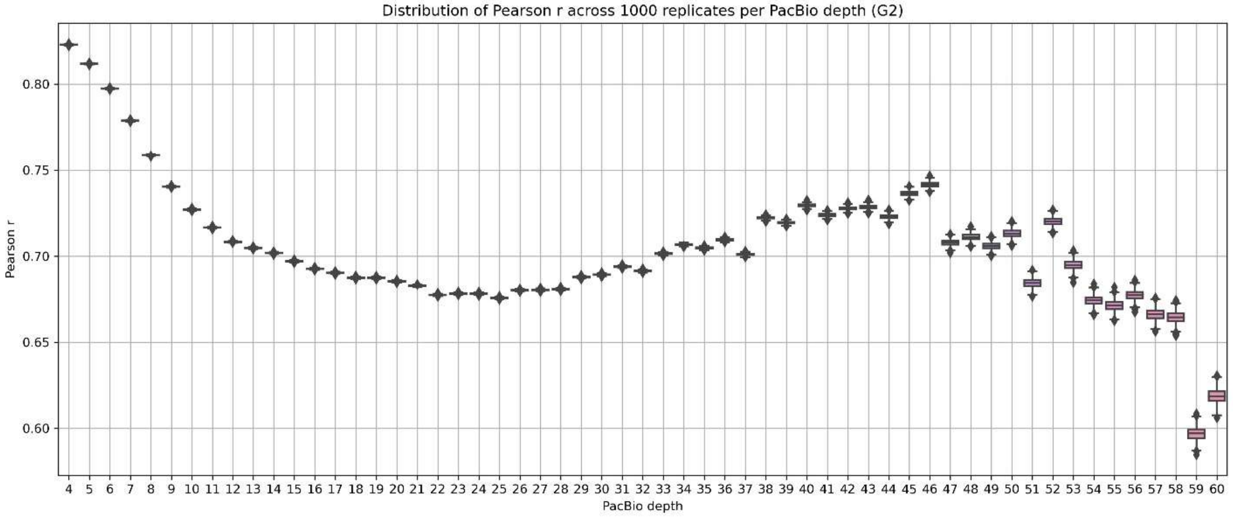

S24 Fig. Methylation concordance between HiFi WGS and WGBS across depth bins after depth-matched downsampling. Downsampling was performed as described in Fig 8. CpG sites were stratified by coverage depth bins. Boxplots show the distribution of Pearson correlation coefficients (r) across 1,000 iterations for each bin. (A) Twin A. (B) Twin B.
