# Supplementary material for: A comparison of DNA methylation detection between HiFi sequencing and whole genome bisulfite sequencing in monozygotic twins with Down syndrome
Source: PLoS One. 2025 Aug 5;20(8):e0329593. doi: 10.1371/journal.pone.0329593 (PMC12324119; doi:10.1371/journal.pone.0329593)
Supplement: Attachment — Submitted filename: response to reviewers.docx [file pone.0329593.s029.docx]

**Dear Editor,**

We sincerely appreciate the reviewers’ thoughtful comments and constructive suggestions, which have greatly helped us improve the clarity and overall quality of our manuscript. We have carefully addressed each point raised and revised the manuscript accordingly. Please find our detailed point-by-point responses below, with corresponding line numbers from the revised manuscript.

Reviewer #1: In this manuscript, the authors assessed CpG methylation concordance between WGBS and HiFi sequencing using monozygotic twins. They found high correlation in CpG methylation results from the two technologies, while also some differences. While the results are interesting, some additional analysis will be beneficial to reveal more results to the readers.

Major points:

1. More QC metrics for either data types, e.g. # of CpGs with different coverage cutoff, % of methylated Cs under CpG, CHH, CHG context, QC for bisulfite conversion efficiency estimated by mC outside of CpG context, etc.

Reply: We thank the reviewer for the suggestions regarding additional quality control metrics. In response, we have expanded our QC reporting as follows:

- We now report the number of CpG sites at each coverage depth for both platforms in **Supplementary Figure S1.**

- For the WGBS dataset, we provide the percentage of methylated cytosines in CpG, CHG, and CHH contexts in **Supplementary Table 2.**

- We evaluated bisulfite conversion efficiency by measuring the proportion of methylated cytosines in non-CpG (CHH) contexts, which serves as an estimate of conversion failure, and report this in **Supplementary Table 2.**

However, for the PacBio HiFi long-read methylation calls, current tools do not support confident context-specific calling in CHH or CHG contexts. Therefore, we restrict CHH/CHG analysis to WGBS only, as noted in the revised manuscript.

We have updated the manuscript accordingly in lines 262-266:

“Bisulfite conversion efficiency was consistently high (97.2–97.36%) across samples and pipelines, confirming effective conversion of unmethylated cytosines. Correspondingly, CpG methylation levels were high (83.7–85.4%), while non-CpG methylation remained low, with CHG and CHH methylation each accounting for only 2.4–2.8% (Supplementary Table 2).”

And in lines 288-301:

“To further assess CpG detection sensitivity, we analyzed CpG site counts across increasing minimum read coverage thresholds (4× to >60×) and plotted cumulative coverage distributions across three datasets: HiFi WGS, wg-blimp, and Bismark. The depth distribution for PacBio HiFi (Supplementary Fig S1A) shows a unimodal and symmetric pattern peaking at 28–30×, indicating relatively uniform coverage. In contrast, both WGBS datasets (Supplementary Fig S1B,C) display right-skewed distributions, with the majority of CpGs covered at low depth (4–10×) and relatively few achieving higher coverage. Over 90% of CpGs in the PacBio HiFi dataset have ≥10× coverage, compared to approximately 65% in the wg-blimp WGBS dataset and under 50% in the Bismark WGBS dataset, as estimated from the cumulative plots (Supplementary Fig S1). Notably, while most CpGs in the WGBS datasets are concentrated at lower depths, the final bin (>60×) includes a small number of CpG sites with extremely high coverage (some exceeding 4000×), which inflate the average coverage values reported in the summary tables and explain the discrepancy between the mean and the more typical coverage levels observed.”

2. Different depth might bias the results. It will be ideal if the author can down-sample the HiFi data to match the WGBS depth;

Reply: We thank the reviewer for pointing out the potential confounding effect of coverage differences between platforms. To address this concern, we initially explored read-level downsampling of PacBio HiFi BAM files. However, this approach proved infeasible due to the nature of long-read sequencing: a single HiFi read typically spans multiple CpG sites, so removing a read to reduce depth at one position would inadvertently affect neighboring sites as well. As a result, it is not practical to control read depth independently at the CpG level using read-based downsampling.

Instead, we implemented a site-level depth-matched subsampling strategy, where we randomly sampled methylated and unmethylated counts at each CpG site. The number of sampled reads was matched to the minimum read depth between PacBio HiFi and WGBS at that site, ensuring fair comparison. This method preserved per-site resolution while eliminating depth as a confounding factor.

We repeated the subsampling process for 1,000 independent replicates across depth levels from 4× to > 60×, and computed Pearson correlation coefficients (r) between platforms in each replicate. The results are shown in Figure 8 and Supplementary Figures S24.

We have updated the Results section accordingly in lines 522–538:

“To further account for coverage-related bias, we implemented a site-level, depth-matched down-sampling strategy for the HiFi WGS and WGBS datasets. At each CpG site, methylation values from the higher-depth platform were randomly down-sampled to match the read depth of the lower-depth platform. This approach enabled the inclusion of a much larger number of CpG sites compared to equal-depth filtering (Fig 7; Supplementary Figure S23). To reduce stochastic variation from random sampling, we performed 1,000 rounds of down-sampling per CpG site and used the average methylation level for downstream analysis. Pearson correlation coefficients were then calculated between the down-sampled HiFi WGS and WGBS methylation levels across various genomic features. The results demonstrated good concordance, with correlation patterns (Fig. 8) that closely resembled those observed in the original (non-downsampled) data (Fig. 4, 5). To further examine the influence of sequencing depth on methylation concordance, we visualized the distribution of Pearson correlation coefficients across 1,000 down-sampling replicates at each PacBio depth level (Supplementary Fig. S24). This analysis provides a complementary view to Figure 8 and reveals a non-linear relationship between depth and concordance. Specifically, correlation values initially decline as depth increases from 4× to approximately 20×, then gradually rise and reach a peak at 46×. Beyond this point, correlation values decline again.”

We also revised the Discussion (lines 635–656) to reflect these insights:

“Building on this, we performed site-level, depth-matched subsampling to equalize coverage between platforms. The overall correlation remained robust, which may be partly due to the effective reduction in read depth introduced by the down-sampling process. The similarity between the down-sampled (Figure 8) and original datasets (Figures 4 and 5) suggests that although absolute coverage influences signal strength, the underlying methylation trends remain robust across technologies.

When we further visualized the distribution of Pearson correlation coefficients across 1,000 depth-matched downsampling replicates (Supplementary Fig. S24), a nonlinear relationship between read depth and inter-platform concordance was observed. Specifically, correlation values initially decreased from 4× to around 20×. At 4–5×, this is likely due to stochastic noise inherent to low-coverage methylation calling, where fractional methylation values are constrained to coarse levels (e.g., 0%, 25%, 50%, 75%, 100%) and therefore have a higher chance of coincidentally matching between platforms, limiting resolution. At 6–20×, predictions may still be influenced by stochastic variability, greater variability in methylation percentage estimates, or insufficient signal accumulation—particularly in regions with low sequence complexity or partial methylation. As coverage increased from 21× to 46×, correlation improved, reflecting greater reliability and precision in methylation estimates as more reads contribute to the aggregated signal. Interestingly, correlation declined again beyond 46×, a pattern we attribute to the small number of CpG sites achieving such high coverage, which introduces sampling noise and reduces statistical robustness. Based on these results, we recommend a minimum coverage of approximately 20× per CpG site for confident methylation quantification using HiFi WGS, balancing both resolution and genomic breadth.”

3. To reveal how depth cutoff affects the correlation, scatter plot between the two technologies colored by coverage in WGBS should be generated.

Reply: We thank the reviewer for this insightful suggestion. As recommended, we investigated the relationship between methylation concordance and WGBS coverage. Initially, we explored a scatter plot colored by WGBS depth; however, this approach resulted in severe overplotting due to the large number of CpG sites, making patterns difficult to interpret.

To address this, we opted to generate two-dimensional binned heatmaps (Figure 7 and Supplementary Figures S23), which allow for clear visualization of CpG density while preserving the correlation structure between WGBS and HiFi methylation levels. Importantly, we stratified the analysis by equal depth levels—restricting to CpG sites with matched coverage between platforms—and grouped them into non-overlapping bins (e.g., 5–10×, 11–15×, etc.).

We have updated the Results section accordingly in lines 631–635:

“Additionally, to account for the prevalence of low-depth CpGs in WGBS, concordance with HiFi WGS was assessed using coverage-matched sites. Concordance increased with coverage, with high-depth CpGs showing strong agreement, while low-depth sites contributed to reduced correlation. A slight decline at the highest-depth bins may reflect increased variability and limited number of matched sites in these extreme ranges.”

We also revised the Discussion (lines 512–522) to reflect these insights:

“To address potential coverage-related bias in methylation concordance, we compared HiFi WGS and WGBS using coverage-matched CpG sites. At lower coverage bins (5–20×), correlations were moderate (r = 0.53–0.65), whereas at higher depths (≥31×), concordance improved substantially (r ≥ 0.72). A decline in correlation was observed in the highest-depth bins (Fig 7 (Twin A) and Supplementary Figure S23 (Twin B)). Although the scatterplots may appear noisy at first glance, this visual impression is primarily driven by a small number of discordant points, as indicated by dense clusters of blue-colored (low-frequency) dots. In contrast, the majority of data points are concentrated along the upper-right diagonal, highlighted by the yellow gradient, indicating strong agreement in methylation levels among coverage-matched CpGs. These results underscore the importance of sufficient sequencing depth for reliable methylation quantification.”

Minor points:

4. To avoid any bias in results of WGBS, another tool such as Bismark should be used to process the data and show no substantial difference from current method.

Reply: We thank the reviewer for this helpful suggestion. To evaluate the robustness of our findings with respect to WGBS processing pipelines, we re-analyzed the data using Bismark, a widely used and well-established bisulfite sequencing analysis tool, as suggested. We then compared the methylation levels from Bismark to HiFi and wg-blimp-based pipeline. The results are shown in Supplementary Figure S2- S11 and Supplementary Table S3.

We have updated the Results section accordingly in lines 312–333:

“Overall, HiFi WGS detected a greater number of mCs while maintaining broadly consistent methylation patterns with WGBS results generated by both the wg-blimp and Bismark pipelines in both twins (Fig. 2–3, Supplementary Figures S2–S3). While the overall trends were preserved across methods—for example, increasing methylation levels from CpG islands to shores and shelves—there were noticeable differences in specific genomic contexts. In particular, methylation profiles between HiFi WGS and Bismark showed greater divergence compared to those between HiFi WGS and wg-blimp. For example, in repetitive elements, HiFi WGS showed higher methylation in SINEs than in LINEs, whereas Bismark displayed the opposite trend (Fig. 2–3, Supplementary Figures S2–S3). Between the two WGBS analysis pipelines, wg-blimp consistently reported a higher number of mCs than Bismark, yet both showed largely concordant patterns across genomic features (Supplementary Figures S4–S5). To further assess the robustness of our methylation threshold, we applied a more stringent cutoff of 80%. The results remained consistent, with over 80% of mCs overlapping those identified using the original 50% threshold (Supplementary Table 4). Overall methylation patterns remained similar across comparisons—HiFi WGS vs. wg-blimp, HiFi WGS vs. Bismark, and wg-blimp vs. Bismark— with the proportion of mCs uniformly decreasing by approximately 10–15% across different genomic regions for all three methods (Supplementary Figures S6–S11). The comparison between the 50% and 80% thresholds also showed consistent trends across HiFi WGS, wg-blimp, and Bismark in most genomic contexts. An exception was observed in CG density categories, where the HiFi WGS profile shifted from a clear decreasing trend under the 50% threshold to a more variable, non-monotonic pattern under the 80% threshold (Fig. 2–3, Supplementary Figures S2–S11).”

And in lines 370-391:

“The distribution patterns in secondary regions were largely consistent between the two techniques for both thresholds (50% and 80%) across various genetic regions including regulatory regions (enhancer and open chromatin) but exhibited variation across certain chromosomes (Fig 3; Supplementary Figure S3, S5, S7, S9, S11). In genetic regions, the lowest proportion of mCs were remarkably indicated in promoters. HiFi WGS remarkably identified more mCs in exons than WGBS (Fig 3A; Supplementary Figure S3A, S5A, S7A, S9A, S11AFig 2D). For regulatory regions, both technologies showed a consistent pattern in which enhancer regions had a slightly higher proportion of mCs compared to open chromatin regions (Fig 3B; Supplementary Figure S3B, S5B, S7B, S9B, S11B). Across all comparisons, the chromosomal distribution of mCs exhibited consistent patterns across detection methods and thresholds, with the exception of chromosomes 15–16, where HiFi WGS showed a slightly elevated proportion of methylated CpGs, diverging from the downward trend observed in both Bismark and wg-blimp (Fig 3C; Supplementary Figure S3C, S5C, S7C, S9C, S11C). HiFi WGS reported the highest mC proportions across all chromosomes, followed by wg-blimp and then Bismark, with all methods showing a pronounced reduction on chromosome Y. Wg-blimp and Bismark produced highly comparable mC levels and profiles across chromosomes. Notably, under the more stringent 80% threshold, the proportion of mCs declined uniformly across chromosomes for each method; however, the relative differences between platforms and the chromosome-specific patterns remained consistent with those observed at the 50% threshold. These findings demonstrate that despite varying detection thresholds and analytical pipelines, the chromosome-level methylation landscape is largely reproducible and biologically meaningful (Fig 3C; Supplementary Figure S3C, S5C, S7C, S9C, S11C).”

We also revised the Discussion (lines 562–563) to reflect these insights:

“Moreover, wg-blimp and Bismark from WGBS data show concordance in methylation pattern with very high correlation (r > 0.9) across genome”

And in lines 570-571:

“Moreover, Bismark tends to report lower CpG depths than wg-blimp, which may be due to differences in alignment stringency, read processing, and CpG counting strategies.”

These results confirm that our conclusions are not dependent on the WGBS processing pipeline, and that Bismark yields results consistent with those obtained from wg-bimp.

5. Annotations with enhancers or open chromatin regions will be helpful.

Answer: In response to the reviewer’s suggestion, we added a new panel (panel B) to Figures 3 and 5, as well as to Supplementary Figures S3, S5, S7, S9, S11, S15, and S17. We used publicly available DNase I hypersensitivity site annotations from ENCODE to identify open chromatin regions, then intersected these regions with CpG sites and computed methylation concordance between platforms.

We have updated the Results section accordingly in lines 376–378:

“For regulatory regions, both technologies showed a consistent pattern in which enhancer regions had a slightly higher proportion of mCs compared to open chromatin regions (Fig 3B; Supplementary Figure S3B, S5B, S7B, S9B, S11B).”

in lines 427-429:

“Both enhancer and open chromatin regions exhibited moderately high methylation levels, with a slight reduction observed in open chromatin (Fig 5B; Supplementary Figure S15B, 17B; leftmost and middle column).”

And in lines 486-488:

“Correlation of methylation levels between detection methods was consistently high across both enhancer and open chromatin (Fig 5B; Supplementary Figures S15B and S17B; rightmost column).”

The Discussion section has been amended to address these findings (lines 613–617):

“Regulatory regions, including enhancers and open chromatin, are typically located in moderate to GC-rich genomic contexts [39, 40, 41]. In our data, these regions exhibited more stable methylation and higher concordance between platforms than low-GC or variable regions, suggesting that their GC content and functional conservation support more reliable methylation detection.”

6. Average signal plot around gene body + promoter will be helpful.

Reply: We thank the reviewer for this suggestion. In response, we generated an average methylation profile aligned to gene architecture, covering 2 kb upstream of the transcription start site (TSS), the entire gene body (scaled to uniform length), and 2 kb downstream of the transcription end site (TES). The resulting plot is presented in Supplementary Figure S18. As expected, both platforms show reduced methylation near the TSS and increased methylation across gene bodies. The overall methylation profiles are highly concordant between platforms, reinforcing the reliability of HiFi-based methylation estimates in both promoter and genic regions.

We have updated the Results section accordingly in lines 449–455.

“In addition, to examine methylation in relation to gene structure, average signal plots were generated spanning 2 kb upstream of the transcription start site (TSS), the scaled gene body, and 2 kb downstream of the transcription end site (TES). All platforms showed a characteristic dip in methylation near the TSS, elevated levels across the gene body, and a slight decline near the TES. The consistency across platforms (HiFi WGS, WGBS-Bismark, and WGBS-MethylDackel) supports the robustness of methylation measurements in genic and promoter regions (Supplementary Figure S18).”

7. How about CHH and CHG context? Can authors also report those regions?

Reply: We thank the reviewer for this suggestion. As follow-up to the quality control metrics reported in response to Point 1, we further examined CHG and CHH methylation levels in different genomic regions using WGBS data. These results are now summarized in Supplementary Figure S19.

We have updated the Results section accordingly in lines 455–457.

“We also investigated non-CpG methylation (CHG and CHH contexts), which showed consistently low levels across all genomic contexts (Supplementary Figure S19).”

8. It will be interesting to perform comparison between the twins to identify differential mCpGs and compare the findings from either technologies to see if any bias present.

Reply: We agree that a comparison of differential methylation between the monozygotic twins using both platforms is scientifically interesting. In fact, this analysis is part of a separate study we are currently preparing, which focuses on integrating multiple variant types (SNV, SV, CNV, STR) along with differential methylation to investigate disease-relevant genomic differences between the twins. For the present manuscript, our primary goal is to evaluate the consistency and concordance of CpG methylation detection between WGBS and HiFi sequencing technologies across the genome. We look forward to sharing the results of the twin-specific comparison within a broader biological framework in a forthcoming publication.

Reviewer #2: I enjoyed reading this manuscript that aims at contracting the methylation landscape between HiFi and WGBS in Down Syndrome patients. While the manuscript was a nice read it needs some additional work to become ready for publication.

Line 45: “pivotal epigenetic genome modification”, remove the word “genome” and let the sentence be “pivotal epigenetic modification”.

Reply: We appreciate the reviewer’s suggestion and have revised the sentence accordingly. The phrase now reads: “pivotal epigenetic modification.”

Materials and Methods: Provide information about the twins, are they male/female, age at recruitment, body composition (height and weight). It can be concluded from your results that they are males because of Y chromosome data, but you still need to say in the method section that they are males.

Reply: We thank the reviewer for this important suggestion. We have updated the Materials and Methods section (lines 123–125) to include relevant demographic and anthropometric details of the twins to clarify this point:

" A pair of male monozygotic twins with trisomy 21 was recruited at 12 years and 9 months of age [14]. The weight and height of the twins at recruitment were 44 kg and 140.6 cm for Twin A, and 45 kg and 141.2 cm for Twin B, respectively.”

Line 115: “the recruitment period spanning from July 18, 2019, to July 17, 2025.” The date is incorrect. We are not in July 2025 yet. Also, if you recruited only a single pair of twins why was it done over a long period?

Reply: We thank the reviewer for pointing this out. The dates refer to the duration of our IRB approval, which spans from July 18, 2019, to July 17, 2025. The twins analyzed in this study were recruited early in the project as part of a broader, ongoing study aimed at identifying rare genetic and epigenetic variants across a range of rare disorders. We have clarified this point in the Materials and Methods section (lines 126–129) to avoid confusion:

"The samples were collected with approval from the institutional review board of Faculty of Medicine of Chulalongkorn University (IRB number 264/62), with the overall recruitment period spanning from July 18, 2019, to July 17, 2025. While the present study focuses on a single pair of twins, the broader study continues to recruit additional participants. These twins were among the earliest participants enrolled in the study."

Line 246 – 248: you say that CpG islands show “demonstrated limited methylated CpG sites”. Is this something commonly reported in Down syndrome cases where CpG islands are not densely methylated? If yes, say something about that and provide a reference. In healthy people it is typical to see unmethylated islands, would that be the case in DS? Also we don’t know the age of the twins, if they are young, then the lack of methylation could be to age and not any other reason.

Reply: We thank the reviewer for this thoughtful comment. We have added a discussion of this point in lines 662–671:

“This pattern is not unique to individuals with DS and has been consistently observed in healthy populations. Although methylation levels can be influenced by age, promoter-associated CpG islands generally remain unmethylated throughout life. Prior studies have shown that in DS, CpG islands largely retain this unmethylated status, with disease-associated methylation changes occurring in a gene-specific rather than global manner —for example, differential methylation of transcription factor genes (e.g., ZNF and HOX families) and CpG islands within the HIST1 gene cluster has been observed in DS patients, potentially affecting chromatin remodeling [47]. These observations suggest that the reduced methylation levels observed in CpG islands in our DS twin samples reflect general epigenomic architecture rather than disease- or age-specific disruption.”

Line 269: “there were a depth of coverage less than” change it so it says: there was a depth of coverage less than”.

Reply: We thank the reviewer for catching this grammatical error. The sentence has been corrected to: “there was a depth of coverage less than”.

Result section: For all correlation combinations, you need to provide scatter plots to show correlation. In those plots, you need to show r value and line of best fit.

Reply: We thank the reviewer for this suggestion. In response, we have generated scatter plots illustrating methylation concordance between WGBS (wg-blimp) and HiFi WGS across various genomic features, including promoters, exons, introns, and repeat classes such as LINE and SINE. Each panel now includes a line of best fit along with the corresponding Pearson correlation coefficient (r) to quantify the agreement. These visualizations are presented in Figure 6 (Twin A) and Supplementary Figure S20 (Twin B). We have also updated the manuscript accordingly, adding the following sentence in lines 488–490:

“Scatter plots (2D binned heatmaps) with linear regression lines showing concordance between WGBS (wg-blimp) and HiFi across different genetic contexts are shown in Fig 6 (Twin A) and Supplementary Figure S20 (Twin B).”

In supp fig 3 and 4, the axes should be labeled. Indicate what the x axis is and what the y axis is.

Reply: Thank you for pointing this out. We have revised the original Supplementary Figures 3 and 4 and updated them as Supplementary Figures S21 and S22 in the Supplementary information. Axis labels have been added for clarity.

In Supplementary Figure S21, the distribution of genetic regions (promoters, exons, introns, UTRs, intergenic regions) is shown across different GC density bins. The x-axis represents GC density categories (20, 40, 60, 80, and 100%), and the y-axis indicates the proportion of regions overlapping each GC density bin.

In Supplementary Figure S22, the x-axis represents individual chromosomes, while the y-axis denotes the proportion of (A) CpG regions, (B) GC density categories, and (C) genetic regions based on the distribution of overlapping CpG sites identified by WGBS (wg-blimp) and HiFi WGS.

Line 419: This is the first time entropy is mentioned in the manuscript. How was entropy measured? There is no mention of this in the method section.

Reply: Thank you for pointing this out. We have now updated the Methods section to include the entropy calculation details (lines 246–254):

**“Sequence Entropy Calculation**

To estimate sequence complexity in different genomic regions, we calculated DNA entropy using the Shannon entropy (H) formula [32]:

$$H=-\sum_{i=1}^{4} p_{i}{log}_{2}p_{i}$$

where $p_{i}$ represents the frequency of each nucleotide (A, T, C, G) in the given region. For each category (tandem repeats, interspersed repeats, and non-repetitive regions), we computed the average entropy across all CpG-containing 100 bp windows overlapping those regions. These values provide a proxy for sequence complexity, with lower entropy indicating more repetitive sequences.”

Discussion

It is not clear what role DS plays in this research. Was it expected that DS would be a major disruptor of methylome? The main purpose of the study is to highlight the differences between HiFi and WGBS. This being done in DS cases is not clear. Was there a reason to think that DS would lead to different methylation readings? Based on the results, the differences between the two methods is based on technology and methodology, and DS plays no role in this difference.

Reply: We thank the reviewer for raising this point. We would like to clarify that Down syndrome (DS) was not selected because we expected it to cause differences in methylation concordance between technologies. Rather, the twins with DS were part of a broader rare disease study that includes genome-wide analysis of SNVs, SVs, CNVs, STRs, and DNA methylation. Given that both short-read and long-read methylation data were available for these samples, we saw an opportunity to perform a direct comparison of methylation measurements across technologies using well-matched biological material.
Importantly, our study does not suggest that DS influences the concordance between HiFi and WGBS; in fact, our results demonstrate that the agreement between technologies is consistent with prior reports in healthy individuals. By including DS samples, our study extends this comparison into a disease-relevant context, supporting the broader utility of long-read methylation profiling in rare disease research.

We have revised the sentence in lines 79–82 to clarify this point:

“While there is no biological rationale to expect that DS would affect the concordance between methylation platforms, the availability of short-read and long-read data provided an opportunity to extend the platform comparison to a disease setting, which has not been systematically explored.”

The authors did not show any concern with the sample size. You only have one pair of DS twins. You need to list that as a limitation.

Reply: We agree with the reviewer that the small sample size is a limitation of this study. We have added the following paragraph in in lines 722-728 to the Discussion section to acknowledge this:

“A key limitation of this study is the small sample size, as the analysis was performed on a single pair of monozygotic twins with Down syndrome. While this provided a unique opportunity to evaluate methylation concordance using matched short- and long-read data in a controlled genetic background, the findings may not fully capture variability across individuals or disease states. Future studies with larger and more diverse cohorts will be important to confirm the generalizability of our observations and further validate the robustness of HiFi methylation profiling across biological contexts.”

Reviewer #3: The stated goal of this project - to compare methylation calling via traditional WGBS with PacBio HiFi sequencing - is a worthwhile undertaking. However, I’m not sure the paper in its current form accomplishes that goal.

The authors argue that a comparison of methylation calling from WGBS and PacBio HiFi sequencing is especially relevant in “complex conditions like Down syndrome”. They state in the Introduction that there “... are still limited studies on the concordance of 5-mC detection across various genomic regions, especially in the context of genetic conditions like Down syndrome.”

From these statements, it seems there are three distinct goals: (1) to compare the two platforms in terms of calling DNA methylation, (2) to compare reproducibility of methylation calls in different genomic contexts, and (3) to study DNA methylation in Down syndrome. To accomplish any of these individual goals would likely require a unique study design. By trying to do many things at once, the current manuscript sacrifices the ability to do any one task sufficiently.

If the goal is to study the role of DNA methylation in down syndrome, the choice of whole blood samples is not well motivated. DNA methylation patterns are cell type specific. It’s not clear how blood cell phenotypes are related to Down syndrome.

If the goal is to compare reproducibility of methylation calls in different genomic contexts, there needs to be a more careful stratification of the genome. We expect that DNA methylation will mark most CpGs in the genome, with the exception being CpG islands, which mark the promoters of most human genes (see e.g. DOI 10.1186/s13072-017-0130-8 10.1038/s41580-019-0159-6). Where the differences in HiFi PacBio sequencing vs WGBS may be most apparent is in highly repetitive regions, such as transposons or pericentromeric regions. A more thorough analysis of this would potentially be informative.

If the goal is to study reproducibility of methylation calls, it is important to control for coverage across the genome. The different coverages for WGBS and HiFi in Table 1 will necessarily lead to differences in methylation calls. The dramatically different read lengths will also introduce bias in methylation interrogation. If the authors want to compare WGBS and HiFi in an unbiased manner, they could sample from the HiFi library to achieve coverage and read lengths analogous to the WGBS libraries and repeat their analysis. In the first paragraph of the results section the authors add that the sequencing depth is not controlled because of “reliance on existing data”. Are these datasets all published?

Reply: We thank the reviewer for their thoughtful assessment and helpful framing of the possible study objectives. We would like to clarify that our study is focused on Objective (1) — to compare methylation calling between WGBS and PacBio HiFi sequencing. We are not investigating the biological role of DNA methylation in Down syndrome (Objective 3), nor are we pursuing reproducibility across genomic contexts as a distinct aim (Objective 2). Rather, we stratified our comparisons by genomic features (e.g., gene annotations, GC content, repetitive elements) to better evaluate how each platform performs across diverse genomic landscapes.

The monozygotic twins with Down syndrome were included because matched short- and long-read sequencing data were available as part of a larger rare disease study. While these samples provide clinical relevance, they were not used to explore DS-specific epigenetic biology. We have revised the Introduction section to more clearly state our objective and prevent this misinterpretation.

We have revised the sentence in lines 101-103 to clarify this point:

“This study focused on assessing the concordance of CpG methylation predictions by stratifying the comparison across various genomic contexts, annotation categories, and sequencing depth levels.”

Regarding the suggestion to match read lengths between HiFi and WGBS libraries, we respectfully note that this would not reflect real-world usage of these platforms. PacBio HiFi sequencing is inherently a long-read technology, and its strength lies in capturing contiguous methylation signals over extended genomic regions — particularly in repetitive and complex areas where short reads struggle. Artificially trimming HiFi reads to resemble short-read libraries would obscure the unique capabilities of long-read methylation profiling and undermine the purpose of evaluating these technologies as they are actually applied in research and clinical settings.

We agree, however, that coverage differences can bias comparisons. To address this, we performed a subsampling analysis to control for depth and assess the stability of concordance across multiple replicate sets. While we initially considered read-level downsampling of PacBio HiFi data, this approach was ultimately not feasible. Due to the nature of long-read sequencing—where a single HiFi read spans multiple CpG sites—removing reads to adjust depth at a specific CpG would unintentionally alter coverage at adjacent sites, making it impractical to control depth precisely at the single-CpG level.

To overcome this limitation, we adopted a site-level, depth-matched subsampling strategy. At each CpG site, we randomly sampled methylated and unmethylated counts from the higher-depth platform to match the lower-depth platform, ensuring a balanced and fair comparison across technologies. This method preserves single-site resolution while mitigating the impact of unequal coverage.

We performed 1,000 rounds of subsampling per site across a range of coverage levels (4× to 60×) and calculated Pearson correlation coefficients (r) for each replicate. The resulting correlation trends are presented in Figure 8 and Supplementary Figure S24.

We have updated the Results section accordingly in lines 522–538:

“To further account for coverage-related bias, we implemented a site-level, depth-matched down-sampling strategy for the HiFi WGS and WGBS datasets. At each CpG site, methylation values from the higher-depth platform were randomly down-sampled to match the read depth of the lower-depth platform. This approach enabled the inclusion of a much larger number of CpG sites compared to equal-depth filtering (Fig 7; Supplementary Figure S23). To reduce stochastic variation from random sampling, we performed 1,000 rounds of down-sampling per CpG site and used the average methylation level for downstream analysis. Pearson correlation coefficients were then calculated between the down-sampled HiFi WGS and WGBS methylation levels across various genomic features. The results demonstrated good concordance, with correlation patterns (Fig. 8) that closely resembled those observed in the original (non-downsampled) data (Fig. 4, 5). To further examine the influence of sequencing depth on methylation concordance, we visualized the distribution of Pearson correlation coefficients across 1,000 down-sampling replicates at each PacBio depth level (Supplementary Fig. S24). This analysis provides a complementary view to Figure 8 and reveals a non-linear relationship between depth and concordance. Specifically, correlation values initially decline as depth increases from 4× to approximately 20×, then gradually rise and reach a peak at 46×. Beyond this point, correlation values decline again.”

We also revised the Discussion (lines 635–656) to reflect these insights:

“Building on this, we performed site-level, depth-matched subsampling to equalize coverage between platforms. The overall correlation remained robust which may be partly due to the effective reduction in read depth introduced by the down-sampling process. The similarity between the down-sampled (Figure 8) and original datasets (Figures 4 and 5) suggests that although absolute coverage influences signal strength, the underlying methylation trends remain robust across technologies.

When we further visualized the distribution of Pearson correlation coefficients across 1,000 depth-matched downsampling replicates (Supplementary Fig. S24), a nonlinear relationship between read depth and inter-platform concordance was observed. Specifically, correlation values initially decreased from 4× to around 20×. At 4–5×, this is likely due to stochastic noise inherent to low-coverage methylation calling, where fractional methylation values are constrained to coarse levels (e.g., 0%, 25%, 50%, 75%, 100%) and therefore have a higher chance of coincidentally matching between platforms, limiting resolution. At 6–20×, predictions may still be influenced by stochastic variability, greater variability in methylation percentage estimates, or insufficient signal accumulation—particularly in regions with low sequence complexity or partial methylation. As coverage increased from 21× to 46×, correlation improved, reflecting greater reliability and precision in methylation estimates as more reads contribute to the aggregated signal. Interestingly, correlation declined again beyond 46×, a pattern we attribute to the small number of CpG sites achieving such high coverage, which introduces sampling noise and reduces statistical robustness. Based on these results, we recommend a minimum coverage of approximately 20× per CpG site for confident methylation quantification using HiFi WGS, balancing both resolution and genomic breadth.”

The datasets used in this study are not yet published, as the sequencing and analysis are part of a broader, ongoing study that integrates multiple variant types—including SNVs, SVs, CNVs, STRs, and differential methylation—to investigate disease-relevant genomic differences between the twins. Short-read data alone were insufficient for comprehensive structural variant discovery, which motivated the use of long-read sequencing. As an added benefit, the long-read platform also provided native DNA methylation measurements, enabling the present comparison. The sequencing depth was not experimentally controlled, as our study was designed around existing data from this larger project.

In addition to these conceptual concerns, I have technical concerns as well:

The wg-blimp analysis pipeline used for the WGBS analysis is not well defended. Utilizing a more standard approach (e.g. Methpipe?) would be more appropriate. One puzzling aspect of the wg-blimp pipeline is the prioritization of quality control _after_ mapping and de-duplication.

Reply: We appreciate the reviewer’s comment regarding the use of the wg-blimp pipeline. We also acknowledge the reviewer’s observation about the ordering of quality control steps in wg-blimp, specifically the placement of QC after mapping and deduplication. This design reflects the pipeline's internal structure and automation of QC reporting, though we agree that the order differs from some conventional workflows.

In response to this concern, and in alignment with a related suggestion from Reviewer 1, we reprocessed the WGBS data using Bismark, a widely recognized and extensively used bisulfite sequencing pipeline. This allowed us to evaluate the reproducibility of our findings using an independent and community-standard tool, thereby addressing potential concerns arising from wg-blimp’s implementation details.

While Methpipe was mentioned as an example, we respectfully note that it is no longer actively maintained. In contrast, Bismark remains under active development and is widely adopted in current methylation studies. We believe that including results from both wg-blimp and Bismark addresses concerns regarding potential pipeline-related biases and supports the robustness of our conclusions. The results are shown in Supplementary Figure S2- S11 and Supplementary Table S3.

We have updated the Results section accordingly in lines 312–333:

“Overall, HiFi WGS detected a greater number of mCs while maintaining broadly consistent methylation patterns with WGBS results generated by both the wg-blimp and Bismark pipelines in both twins (Fig. 2–3, Supplementary Figures S2–S3). While the overall trends were preserved across methods—for example, increasing methylation levels from CpG islands to shores and shelves—there were noticeable differences in specific genomic contexts. In particular, methylation profiles between HiFi WGS and Bismark showed greater divergence compared to those between HiFi WGS and wg-blimp. For example, in repetitive elements, HiFi WGS showed higher methylation in SINEs than in LINEs, whereas Bismark displayed the opposite trend (Fig. 2–3, Supplementary Figures S2–S3). Between the two WGBS analysis pipelines, wg-blimp consistently reported a higher number of mCs than Bismark, yet both showed largely concordant patterns across genomic features (Supplementary Figures S4–S5). To further assess the robustness of our methylation threshold, we applied a more stringent cutoff of 80%. The results remained consistent, with over 80% of mCs overlapping those identified using the original 50% threshold (Supplementary Table 4). Overall methylation patterns remained similar across comparisons—HiFi WGS vs. wg-blimp, HiFi WGS vs. Bismark, and wg-blimp vs. Bismark— with the proportion of mCs uniformly decreasing by approximately 10–15% across different genomic regions for all three methods (Supplementary Figures S6–S11). The comparison between the 50% and 80% thresholds also showed consistent trends across HiFi WGS, wg-blimp, and Bismark in most genomic contexts. An exception was observed in CG density categories, where the HiFi WGS profile shifted from a clear decreasing trend under the 50% threshold to a more variable, non-monotonic pattern under the 80% threshold (Fig. 2–3, Supplementary Figures S2–S11).”

And in lines 370-391:

“The distribution patterns in secondary regions were largely consistent between the two techniques for both thresholds (50% and 80%) across various genetic regions including regulatory regions (enhancer and open chromatin) but exhibited variation across certain chromosomes (Fig 3; Supplementary Figure S3, S5, S7, S9, S11). In genetic regions, the lowest proportion of mCs were remarkably indicated in promoters. HiFi WGS remarkably identified more mCs in exons than WGBS (Fig 3A; Supplementary Figure S3A, S5A, S7A, S9A, S11AFig 2D). For regulatory regions, both technologies showed a consistent pattern in which enhancer regions had a slightly higher proportion of mCs compared to open chromatin regions (Fig 3B; Supplementary Figure S3B, S5B, S7B, S9B, S11B). Across all comparisons, the chromosomal distribution of mCs exhibited consistent patterns across detection methods and thresholds, with the exception of chromosomes 15–16, where HiFi WGS showed a slightly elevated proportion of methylated CpGs, diverging from the downward trend observed in both Bismark and wg-blimp (Fig 3C; Supplementary Figure S3C, S5C, S7C, S9C, S11C). HiFi WGS reported the highest mC proportions across all chromosomes, followed by wg-blimp and then Bismark, with all methods showing a pronounced reduction on chromosome Y. Wg-blimp and Bismark produced highly comparable mC levels and profiles across chromosomes. Notably, under the more stringent 80% threshold, the proportion of mCs declined uniformly across chromosomes for each method; however, the relative differences between platforms and the chromosome-specific patterns remained consistent with those observed at the 50% threshold. These findings demonstrate that despite varying detection thresholds and analytical pipelines, the chromosome-level methylation landscape is largely reproducible and biologically meaningful (Fig 3C; Supplementary Figure S3C, S5C, S7C, S9C, S11C).”

We also revised the Discussion (lines 562–563) to reflect these insights:

“Moreover, wg-blimp and Bismark from WGBS data show concordance in methylation pattern with very high correlation (r > 0.9) across genome.”

And in lines 570-571:

“Moreover, Bismark tends to report lower CpG depths than wg-blimp, which may be due to differences in alignment stringency, read processing, and CpG counting strategies.”

These results confirm that our conclusions are not dependent on the WGBS processing pipeline, and that Bismark yields results consistent with those obtained from wg-bimp.

There are several seemingly arbitrary thresholds introduced in the manuscript. For example, regarding CpGs as methylated with methylation level is >= 50 with coverage >= 4 is not justified. Generally DNA methylation profiles follow a bimodal distribution with the largest mode corresponding to CpGs being methylated and another mode corresponding to unmethylated. Do the methylation values for HiFi and WGBS here follow that pattern? That could be used to set thresholds.

Reply: We thank the reviewer for highlighting the importance of threshold selection. We used a minimum coverage threshold of 4× based on the default recommendation of PacBio’s CpG methylation calling tools. For the methylation level threshold, we applied a 50% cutoff to distinguish between methylated and unmethylated CpGs, as this value represents a statistically neutral midpoint and aligns with interpretations used in several methylation analysis tools and community standards. To further justify this choice, we examined the distribution of methylation levels for both HiFi WGS and WGBS data (Response Figure 1). As shown, the distributions exhibit a bimodal pattern, with a minor peak near 0% and a dominant peak near 90–100% methylation, consistent with typical methylation profiles in human somatic cells. Based on these distributions, we also evaluated a more stringent threshold at 80%, given the sharp increase in CpG counts beginning around that level. The corresponding results are provided in Supplementary Table 4 and Supplementary Figures S6–S11.

We have updated the Results section accordingly (lines 323–333).

“To further assess the robustness of our methylation threshold, we applied a more stringent cutoff of 80%. The results remained consistent, with over 80% of mCs overlapping those identified using the original 50% threshold (Supplementary Table 4). Overall methylation patterns remained similar across comparisons—HiFi WGS vs. wg-blimp, HiFi WGS vs. Bismark, and wg-blimp vs. Bismark— with the proportion of mCs uniformly decreasing by approximately 10–15% across different genomic regions for all three methods (Supplementary Figures S6–S11). The comparison between the 50% and 80% thresholds also showed consistent trends across HiFi WGS, wg-blimp, and Bismark in most genomic contexts. An exception was observed in CG density categories, where the HiFi WGS profile shifted from a clear decreasing trend under the 50% threshold to a more variable, non-monotonic pattern under the 80% threshold (Fig. 2–3, Supplementary Figures S2–S11).”

And in lines 385-391:

“Notably, under the more stringent 80% threshold, the proportion of mCs declined uniformly across chromosomes for each method; however, the relative differences between platforms and the chromosome-specific patterns remained consistent with those observed at the 50% threshold. These findings demonstrate that despite varying detection thresholds and analytical pipelines, the chromosome-level methylation landscape is largely reproducible and biologically meaningful (Fig 3C; Supplementary Figure S3C, S5C, S7C, S9C, S11C)”

(A)	(B)

(C)	(D)

**Response Figure 1. Distribution of CpG methylation levels in HiFi WGS and WGBS datasets.**
Histograms show the distribution of methylation levels (0–100%) for CpG sites in (A) HiFi WGS Twin A, (B) HiFi WGS Twin B, (C) WGBS Twin A, and (D) WGBS Twin B.
